# Ectopic overexpression of *ShCBF1* and *SlCBF1* in tomato suggests an alternative view of fruit responses to chilling stress postharvest

**DOI:** 10.3389/fpls.2024.1429321

**Published:** 2024-08-05

**Authors:** Karin Albornoz, Jiaqi Zhou, Florence Zakharov, Jonas Grove, Minmin Wang, Diane M. Beckles

**Affiliations:** Department of Plant Sciences, University of California Davis, Davis, CA, United States

**Keywords:** postharvest chilling injury, *Solanum lycopersicum*, C-binding factor (CBF1), chilling acclimation, cold response, fruit senescence

## Abstract

Postharvest chilling injury (PCI) is a physiological disorder that often impairs tomato fruit ripening; this reduces fruit quality and shelf-life, and even accelerates spoilage at low temperatures. The *CBF* gene family confers cold tolerance in *Arabidopsis thaliana*, and constitutive overexpression of *CBF* in tomato increases vegetative chilling tolerance, in part by retarding growth, but, whether CBF increases PCI tolerance in fruit is unknown. We hypothesized that *CBF1* overexpression (OE) would be induced in the cold and increase resistance to PCI. We induced high levels of *CBF1* in fruit undergoing postharvest chilling by cloning it from *S. lycopersicum* and *S. habrochaites*, using the stress-inducible *RD29A* promoter. Harvested fruit were cold-stored (2.5°C) for up to three weeks, then rewarmed at 20°C for three days. Transgene upregulation was triggered during cold storage from 8.6- to 28.6-fold in *SlCBF1*-OE, and between 3.1- to 8.3-fold in *ShCBF1*-OE fruit, but developmental abnormalities in the absence of cold induction were visible. Remarkably, transgenic fruit displayed worsening of PCI symptoms, i.e., failure to ripen after rewarming, comparatively higher susceptibility to decay relative to wild-type (WT) fruit, lower total soluble solids, and the accumulation of volatile compounds responsible for off-odors. These symptoms correlated with *CBF1* overexpression levels. Transcriptomic analysis revealed that the ripening and biotic and abiotic stress responses were altered in the cold-stored transgenic fruit. Seedlings grown from ‘chilled’ and ‘non-chilled’ WT fruit, in addition to ‘non-chilled’ transgenic fruit were also exposed to 0°C to test their photosynthetic response to chilling injury. Chilled WT seedlings adjusted their photosynthetic rates to reduce oxidative damage; ‘non-chilled’ WT seedlings did not. Photosynthetic parameters between transgenic seedlings were similar at 0°C, but *SlCBF1*-OE showed more severe photoinhibition than *ShCBF1*-OE, mirroring phenotypic observations. These results suggest that 1) *CBF1* overexpression accelerated fruit deterioration in response to cold storage, and 2) Chilling acclimation *in fructus* can increase chilling tolerance in seedling progeny of WT tomato.

## 1 Introduction

Refrigeration is indisputably the most effective strategy to prolong shelf-life, preserve quality and delay the deterioration of many fruits and vegetables (Kader, 2002). However, in cold-sensitive commodities such as tomato (*Solanum lycopersicum* L.), storage at temperatures between 0-12°C induces the onset of molecular, biochemical, and physiological alterations known as postharvest chilling injury (PCI), which are manifested when fruit are rewarmed to room temperature (Lyons, 1973).

PCI is a complex and multilayered phenomenon. Its early stages are temperature-dependent and are mediated by physical changes in cellular membranes (Luengwilai et al., 2018). Loss of membrane stability triggers the activation of a signal transduction cascade that transmits the cold stimulus downstream through a series of molecular players, e.g., second messengers, eliciting symptoms characteristic of this disorder (Lyons, 1973). These symptoms include modifications in respiration and ethylene production, disruption in the synthesis of aroma volatiles, accumulation of reactive oxygen species (ROS), lipid peroxidation, and DNA and protein damage (Albornoz et al., 2022). These molecular and cellular processes ultimately lead to failures in fruit ripening, the development of surface pitting, seed browning and higher susceptibility to postharvest decay (Moline, 1976; Jackman et al., 1989; Biswas et al., 2016).

Most of what is known about the cold signal transduction pathway in plants, comes from studies of *Arabidopsis thaliana*, which is able to cold-acclimate and endure freezing temperatures (Gilmour et al., 1988). The *C*-Binding Factor (*CBF*) gene family (*AtCBF*1-3) of transcription factors are positive regulators of the cold response that interact with the *cis*-elements of downstream cold-responsive genes (Thomashow et al., 2001; Thomashow, 2010). These target genes, also known as the CBF-regulon, encode protective proteins and enzymes, and are involved in the synthesis of metabolites that enhance the plant’s fitness during cold stress (Thomashow et al., 2001; Thomashow, 2010).

In tomato fruit, *SlCBF1-3* genes are also induced by cold (Zhao et al., 2009a; Zhang et al., 2016; Albornoz et al., 2019), but the size and types of genes comprising the CBF regulon are not the same as in Arabidopsis (Zhang et al., 2016). This might partly explain tomato’s inability to cold acclimate (Zhang et al., 2004). Different members of the Arabidopsis *CBF* gene family have been ectopically expressed in tomato plants under the control of the constitutive *CaMV35S* promoter, resulting in increased stress tolerance, but with concomitant growth reduction and flowering delay (Hsieh et al., 2002a, 2002; Zhang et al., 2004). This, due to CBF’s involvement in gibberellin (GA) repression, and DELLA protein accumulation (Achard et al., 2008; Zhou et al., 2017). A transgenic phenotype overexpressing *AtCBF1* in tomato fruit was characterized, and revealed this gene influenced ripening as well as fruit’s response to postharvest cold stress (Albornoz et al., 2023).

In this work, we hypothesized that *CBF1* overexpression in tomato cv. Micro-Tom fruit during postharvest chilling would enhance fruit tolerance to cold stress and reduce the incidence of PCI. We cloned this gene from two sources: cultivated tomato (*SlCBF1*) and the wild-tomato relative *Solanum habrochaites* (*ShCBF1*). *S. habrochaites* has been extensively studied due to its tolerance to cold stress (Patterson et al., 1984; Goodstal et al., 2005; Cao et al., 2014). *ShCBF1* has been cloned and expressed into Arabidopsis plants conferring tolerance to freezing and salinity but displaying phenotypic abnormalities (Li et al., 2014). In this study, both *ShCBF1* and *SlCBF1* genes were driven by the stress-inducible promoter *RD29A* (Yamaguchi-Shinozaki and Shinozaki, 1994). Our goal was to specifically induce *CBF1* expression in harvested fruit stored in the cold, as well as minimize pleiotropic effects caused by constitutive overexpression.

To test our hypothesis, transgenic fruit were cold-stored, which elicited ectopic *CBF* expression, and their postharvest performance was examined and compared to wild-type fruit under the same conditions. To broaden the scope of traditional studies of chilling injury beyond fruit postharvest, we also tested if the photosynthetic responses of *CBF1*-overexpressing seedlings would be affected by cold stress. The goal was to understand the physiological effects of additional *CBF1* transcripts at different phases of the plant life cycle. Finally, we evaluated the influence of postharvest fruit chilling on seeds and seedling traits that were measured under control (room temperature) or cold conditions, to explore the concept of *transgenerational* adaptive mechanisms transmitted from fruit to progeny.

## 2 Materials and methods

### 2.1 Construct development

**Figure 1:**
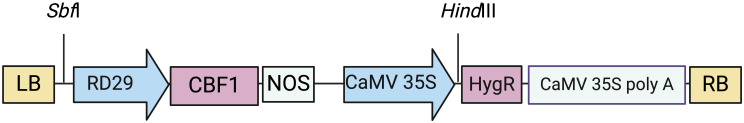
The base constructs used for plant transformation. In this experiment, the *CBF1* was cloned from *S.lycopersicum*, i.e., *pCAMBIA-RD29A*::*SlCBF1* or from *S.habrochaites*, i.e., *pCAMBIA-RD29A*::*ShCBF1.* Elements from left to right: LB, Left T-DNA Border; *RD29A* pro, stress-inducible promoter from *A. thaliana*; *SlCBF1 or ShCBF1*, C-binding factor from either *S. lycopersicum* or *S. habrochaites*; NOS ter, terminator sequence; 35S, *CaMV 35S* promoter; HygR, hygromycin resistance gene; *CaMV 35S* poly A signal, termination signal; RB, right T-DNA Border. Vertical lines represent restriction sites.

The stress-inducible promoter *RD29A* (GenBank Accession no. CS191722.1), the *ShCBF1* gene (GenBank Accession no. KX890304.1) and the *SlCBF1* gene (GenBank Accession no. NM_001247194.2) were separately cloned from *Arabidopsis thaliana* Col-0 genomic DNA, *S. lycopersicum* cv. Micro-Tom genomic DNA and *S. habrochaites* LA1777 (The C.M. Rick Tomato Genetics Resource Center at University of California, Davis) genomic DNA, respectively (**Supplementary Table S1**). The *RD29A* promoter and *CBF1* genes were amplified separately, and the *RD29A* reverse primer was designed to include a 20 bp overlapping sequence with the forward primer of *ShCBF1 or SlCBF1.* A second PCR reaction was set up using the products of the first reactions as templates. The resulting assembly PCR product consisted of *RD29A* located at the 5’ end, having a *SbfI* restriction site and *ShCBF1* or *SlCBF1* at the 3’ end, having a *Hin*dIII restriction site. These products were cloned into pCAMBIA1300 *Sbf*I and *Hin*dIII sites, and the absence of mutations was confirmed by sequencing. The resulting constructs were named *pCAMBIA-RD29A*::*ShCBF1* and *pCAMBIA1300-RD29A*::*SlCBF1* (**Figure 1**). The amino acid sequences of *ShCBF1* and the native *SlCBF1* gene share a 92% identity according to the Multiple Sequence Alignment by CLUSTALW tool (Kyoto University Bioinformatics Center). Constitutive overexpression of *AtCBF1* (Hsieh et al., 2002a, 2002; Zhang et al., 2011) or *AtCBF3* (Zhang et al., 2004) in tomato plants has been studied by others, therefore the amino acid alignment and phylogenetic relationships between these, and *ShCBF1* and *SlCBF1* proteins, are shown in **Supplementary Figure S6**.

### 2.2 Plant transformation and growing conditions

Tomato (*Solanum lycopersicum* L. cv. Micro-Tom) was chosen based on its short life cycle, ease of transformation and small size at full maturity (Meissner et al., 1997). The latter allowed growing a high density of plants, critical in postharvest studies, which require harvesting numerous fruit at the same maturity stage. Micro-Tom cotyledons and hypocotyls were transformed with *Agrobacterium tumefaciens* EHA105 cultures carrying either *pCAMBIA-RD29A::ShCBF1* or *pCAMBIA-RD29A::SlCBF1* constructs. Hygromycin B (15 mg/L) was used as selection agent, and transformants were confirmed by PCR. Rooted plants (T_0_ generation) were transferred to a greenhouse at UC Davis and grown at temperatures between 25-30°C. Two independent transformation events per construct were used in this study: ‘Sh-13’ (T_2_ generation) and ‘Sh-36’ (T_2_ generation) for *pCAMBIA-RD29A::ShCBF1* (*ShCBF1*-OE lines), and ‘Sl-2’ (T_3_ generation) and ‘Sl-12’ (T_2_ generation) for *pCAMBIA-RD29A::SlCBF1* (*SlCBF1*-OE lines). A null-segregant (‘NS’), a transformant that is negative for the transgene after segregation, was used as a genetic control, and was denoted here as the wild-type (‘WT’) event.

### 2.3 Transgene copy number determination

Genomic DNA isolation (Fulton et al., 1995) was carried out on two seedlings from each of the transgenic genotypes Sh-13 and Sl-2 using the *Prosystemin* (*Prosys*) gene as an endogenous control as previously reported (Albornoz et al., 2023). Primers were designed based on a conserved region between *ShCBF1* and *SlCBF1* (**Supplementary Table S1**). Transgene copy number was calculated by Real-Time Quantitative PCR (RT-qPCR) as the ratio of the copy number of *SlCBF1* or *ShCBF1* to *Prosys*, respectively (Shepherd et al., 2009).


$$Ratio=\frac{1+Efficiency_{CBF1}{\;}^{Ct\;CBF1}}{1+Efficiency_{Prosys}{\;}^{Ct\;Prosys}}$$

### 2.4 Fruit harvest and phenotypic characterization

#### 2.4.1 Fruit shape index

The width and height of 100 fruit per genotype were recorded. Fruit shape index (FSP) was determined as the ratio of maximum height to maximum width. When FSP = 1, this indicated a round fruit. In contrast, when FSP > 1 or FSP< 1, this indicates an elongated or squat fruit, respectively (Brewer et al., 2006).

#### 2.4.2 Postharvest storage preparation

**Figure 2:**
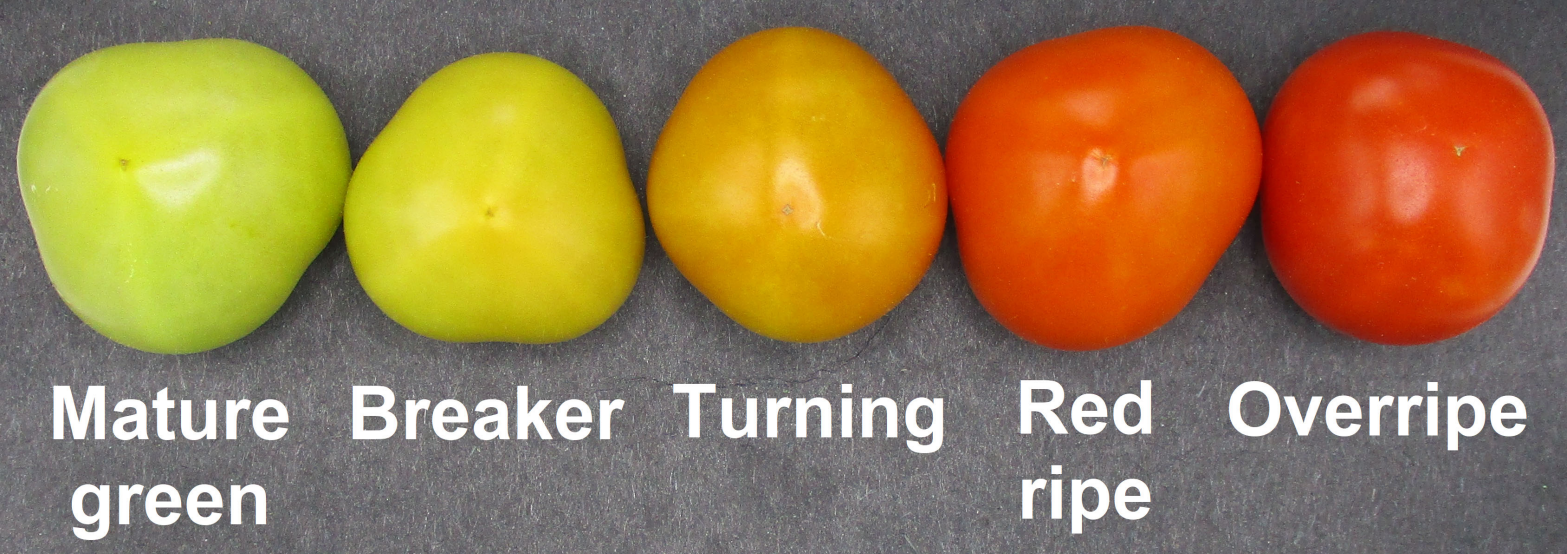
Maturity stages of Micro-Tom tomato.

Fruit were randomly harvested from a group of 50 WT plants, 80 Sh-13 and Sl-2 plants, 26 Sh-36 plants and 76 Sl-12 individuals. Unblemished fruit were harvested at breaker stage (**Figure 2**), soaked in 0.25% (v/v) sodium hypochlorite for three minutes and gently blotted until dry with paper towels. For cold storage phenotyping, Sh-13, Sh-36, Sl-2, Sl-12, and WT fruit were stored at 2.5°C for one, two or three weeks, or transferred to 20°C for three days (‘rewarming’ or ‘RW’) to induce PCI. Characterization of fruit at 20°C was carried out for genotypes Sh-13, Sl-2, and WT for up to 14 days.

#### 2.4.3 Chilling injury index (CII) and decay incidence

Fruit were removed from the 2.5°C room after one, two or three weeks of storage, transferred to 20°C for three days, and evaluated for CII based on the development of surface pitting and the presence of decay. Pitting severity was determined visually on each fruit based on the percentage of surface affected using a five-point scale as follows: 0 = no pitting, 1 =< 25%, 2 = 25-50%, 3 = 50-75%, 4 = > 75% (Aghdam et al., 2014). CII was calculated using the formula:


$$\%\;CII=\frac{\sum [(CII\;level)\;x\;(\#fruit\;at\;that\;CII\;level)]}{4\;x\;total\;\#\;fruit}x\;100$$

**Table 1** shows the total fruit evaluated for each treatment.

**Table 1:** Sample size for Chilling injury index and decay of *CBF1*-OE lines, and wild-type (‘WT’) tomato fruit.

Genotype	1w+3d	2w+3d	3w+3d
**WT**	83	114	118
**Sh-13**	122	100	62
**Sh-36**	68	55	94
**Sl-2**	21	60	36
**Sl-12**	26	54	123

After storage at 2.5°C for 1 (‘1w+3d’), 2 (‘2w+3d’) or 3 (‘3w+3d’) weeks, fruit were transferred to 20°C for 3 days.

#### 2.4.4 Color determination

The Hue angle was used as a descriptor and recorded with a Konica Minolta colorimeter (Chroma Meter CR-400, Konica Minolta Sensing Americas, Ramsey, NJ, USA). A 2° observer and standard illuminant C setting in the L* a* b* scale was utilized. A total of 40 fruit per time point for each genotype were used. Lower Hue values are indicators of redder (riper) fruit, whereas higher values represent greener fruit. **Table 2** shows the total fruit evaluated for each treatment.

**Table 2:** Sample size for color (Hue angle) of *CBF1*-OE lines, and wild-type (‘WT’) tomato fruit.

Genotype	0w	1w	1w+3d	2w	2w+3d	3w	3w+3d
**WT**	86	101	83	118	118	120	120
**Sh-13**	69	126	126	85	105	62	62
**Sh-36**	65	86	68	55	55	56	94
**Sl-2**	67	63	52	63	63	36	36
**Sl-12**	67	24	24	36	54	60	127

Fruit were stored at 2.5°C for 1 (‘1w+3d’), 2 (‘2w+3d’) or 3 (‘3w+3d’) weeks, or followed by transfer to 20°C for 3 days.

#### 2.4.5 Total soluble solids

The percentage of soluble solids was determined in Sh-13, Sl-2 and WT fruit juice using a digital refractometer HI-96801 (Hanna Instruments, Rhode Island, United States) in a total of 18 fruit per treatment for each genotype.

#### 2.4.6 Respiration and ethylene evolution rates

Fruit from genotypes Sh-13, Sl-2 and WT were placed in 450 mL-jars connected to flow boards using capillary tubes to control flow rates. A total of six biological replicates were used for each of the genotypes. Each biological replicate corresponded to a jar containing 22 fruit. A total of 10 mL of gas samples for carbon dioxide (CO_2_) and ethylene were taken with a plastic syringe. Respiration rates were measured by injecting the gas sample into a high-range CO_2_ analyzer (Horiba VIA-510, Kyoto, Japan). A standard of 0.25% (v/v) CO_2_ was used for calibration. Ethylene concentration was measured with a gas chromatograph (Model 211, AGC Series 400; Hach-Carle Co., Colo., U.S.A.). Standards of 100 ppb or 1 ppm ethylene were used for calibration. All measurements took place at the corresponding storage temperatures (2.5 or 20°C) daily.

#### 2.4.7 Volatile compounds analysis by solid phase microextraction (SPME) coupled with gas chromatography -mass spectrometry (GC-MS)

Breaker fruit from lines Sh-13, Sl-2, and WT stored at 2.5°C for 2 weeks, and RW for 3 days were removed from storage and kept at room temperature for 16 h before processing for volatile extraction. Tomato fruit were mixed with saturated calcium chloride solution (1:1, w/v) containing 2 μM 3-octanone as an internal standard, and then blended in a commercial blender for 1 min. Four milliliters of slurry of each sample were transferred into a 20 mL amber headspace vial, exposed to a SPME fiber (Supelco, USA), and stored at -80°C until analyzed on a GC-MS unit (Wang et al., 2019), except that a DB-WAX column (30 m x 0.25 mm, film thickness 0.25 μm) and a different oven program was used. The initial oven temperature was set at 40°C for 5 min, followed by an increase to 80°C at a rate of 5°C min^-1^, then an increase to 200°C at a rate of 10°C min^-1^, and another increase to 250°C at a rate of 20°C min^-1^. The AMDIS software (version 2.64) was used for spectral deconvolution (Stein, 1999) coupled with SpectConnect to consolidate the data (Styczynski et al., 2007). Compound identity was queried with NIST library (v. 2.0) and confirmed by matching the retention time in ChemStation (E.02.02.1431, Agilent, Santa Clara, CA, USA). A total of three biological replicates were used for each of the genotypes. Each biological replicate consisted of ~ 15 g of tomato fruit.

### 2.5 RNA isolation

Total RNA was isolated (Wang et al., 2009) from 100 mg of frozen fruit or leaf powder, depending on the experiment, and treated with the DNase TURBO DNA free Kit (Life Technologies, Carlsbad, CA, USA). RNA integrity and purity were assessed by agarose gel electrophoresis, and the A260/A280 and A260/A230 ratios.

### 2.6 Real-time quantitative PCR

Copy DNA (cDNA) was synthesized from 1 µg RNA. Cycling conditions for cDNA synthesis and RT-qPCR were the same as described in previous reports (Albornoz et al., 2019). Primers were designed based on the cDNA sequences available on GenBank (**Supplementary Table S1**).

The reaction efficiency was calculated through the standard curve method, using a cDNA dilution series (Taylor et al., 2010). The specificity of the primers was validated by melt-curve analysis (Taylor et al., 2010). The Pfaffl method was used for data normalization and relative quantification of transcript production (Pfaffl, 2012). A reference gene, actin (*SlACT7*) was used as the control for data normalization. Three biological and three technical replicates were used. The pericarp region of six fruit, or one true leaf per seedling corresponded to a biological replicate.

### 2.7 RNA sequencing

#### 2.7.1 Messenger RNA isolation

Assessment was done on breaker fruit from lines Sh-13, Sl-2, and WT stored at 2.5°C for six hours or 1 week. Two biological replicates were used per genotype. A biological replicate consisted of the pericarp region of six fruit. The mRNA was isolated from 5 µg total RNA using the NEBNext^®^ Poly(A) mRNA Magnetic Isolation Module.

#### 2.7.2 3’ DGE RNA-Seq library construction and sequencing

Each treatment included two biological replicates. In total, 12 libraries were constructed using Strand-specific mRNA-library prep kits (Amaryllis Nucleics, Oakland, CA). Library concentration was assessed by a Qubit™ device (Invitrogen, Waltham, MA, USA), and the quality was checked by a bioanalyzer, following HiSeq PE150 sequencing in Novogene Co. (Beijing, China). The raw sequencing reads were trimmed according to quality score by FastQC (Andrews, 2010). The reads alignment was processed by the STAR software (Dobin et al., 2013) based on the tomato reference genome SL4.0 (Sol Genomics Network). The gene read counts were generated using the HTSeq package (Putri et al., 2022). Differentially expressed genes (DEGs) were extracted using the DEseq2 tool (Love et al., 2014). The Gene ontology (GO) functional enrichment analyses of DEGs were performed using the g:Profiler online tool, genome assembly SL3.0, accession GCA_000188115.

### 2.8 Vegetative stage tests

The effect of fruit storage conditions on the performance of seed and seedling progeny was investigated. Tests of percent germination, early growth and photosynthetic response in the WT and transgenic seeds, or seedlings obtained from PCI-affected or control fruit, were performed.

#### 2.8.1 CBF1 expression in cold-exposed transgenic and wild-type seedlings

Three seedlings from the wild-type, and from transgenic Sh-13 and Sl-2 lines obtained from non-chilled fruit were grown until cotyledons were fully expanded, transplanted into a 36-cell polyethylene growth tray containing UC Mix substrate and transferred to the greenhouse until plants were at the six-(wild-type) or five-(transgenic) leaf stage. Plants were transferred to a room at 0°C for up to 24 hours. Leaf tissue was excised from each plant, and *CBF1* expression was measured by RT-qPCR.

#### 2.8.2 Seed color, germination, and seedling features

Seeds were collected from WT fruit that were harvested at breaker but stored at two conditions: a) room temperature until fully ripe (‘non-chilled’) or, b) 2.5°C for three weeks, followed by transfer to room temperature until fully ripe (‘chilled’).

Color (L* value or ‘lightness’) of dry seeds was recorded using a Konica Minolta colorimeter according to 2.4.4. When L* value = 0, it indicates darkness or absence of light (black), whereas when L* = 100, it represents the brightest white. Three replicates were used, each consisting of approximately 150 seeds. Seed weight was recorded, and three replicates were used, each consisting of a group of 50 dry seeds.

Seeds were soaked in 2.7% (v/v) sodium hypochlorite for one hour, rinsed thoroughly in running water, placed into Petri dishes with damp paper towels and located in a 20°C (± 2°C) room under 16/8 hour-photoperiod for one week. The number of germinated seeds from each type was recorded after one week to determine the germination percentage. Hypocotyls (‘shoots’) and roots were excised, and their fresh weight was recorded. Three replicates, each representing a Petri dish with 20 seeds for each type, were used.

#### 2.8.3 Photosynthetic performance of seedlings under stress

Sixteen seedlings from the wild-type (‘chilled’ and ‘non-chilled’), and from transgenic Sh-13 and Sl-2 lines were grown as indicated in 2.8.1. Seedlings were placed at 2.5 or 0°C under a 16/8 photoperiod. Photosynthetic parameters: LEF (Linear Electron Flow), Phi2 (efficiency of Photosystem II, PSII), NPQt and PhiNPQ (estimates of Non-Photochemical Quenching), PhiNO (estimate of incoming light lost in non-regulated processes that can induce photodamage), relative chlorophyll, and PAR (Photosynthetically Active Radiation) were measured on the 4^th^ leaf of each plant after 24, 48 and 72 hours with a MultispeQ device (PhotosynQ LLC, East Lansing, MI, USA) (Kuhlgert et al., 2016).

### 2.9 Statistical analysis

Experiments were carried out with a complete randomized design. Statistical analysis was performed using SAS software version 9.4 or Microsoft Excel. To detect significant differences between treatments, analysis of variance (ANOVA) or unpaired *t*-test (with either equal or unequal variance) were conducted. For mean comparison, Tukey’s test was used (α = 0.05). The *z*-score test for two population proportions was used to determine if CII values and decay, and germination % between treatments, differed significantly (*p* < 0.05). *p*-values were symbolized as follow: *p* < 0.05 (*), *p* ≤ 0.01 (**) and *p* ≤ 0.001 (***). Standard error (SE) was used as a measure of data variability.

## 3 Results

### 3.1 Transgenic plant and fruit characterization

The growth and development of the transgenic lines were altered by the expression of *CBF1*. Germination percentages were significantly reduced, and seedlings displayed high mortality (data not shown), suggesting that transgene homozygosis may be associated with a lethal phenotype. The inconclusive results from the transgene copy number estimation in lines Sh-13 and Sl-2 support this observation. *ShCBF1*-OE and *SlCBF1*-OE were stunted, with shorter internodes, and the leaves underwent accelerated senescence, produced less inflorescences, and consequently, less fruit, than wild-type individuals (**Supplementary Figure S4**).

**Figure 3:**
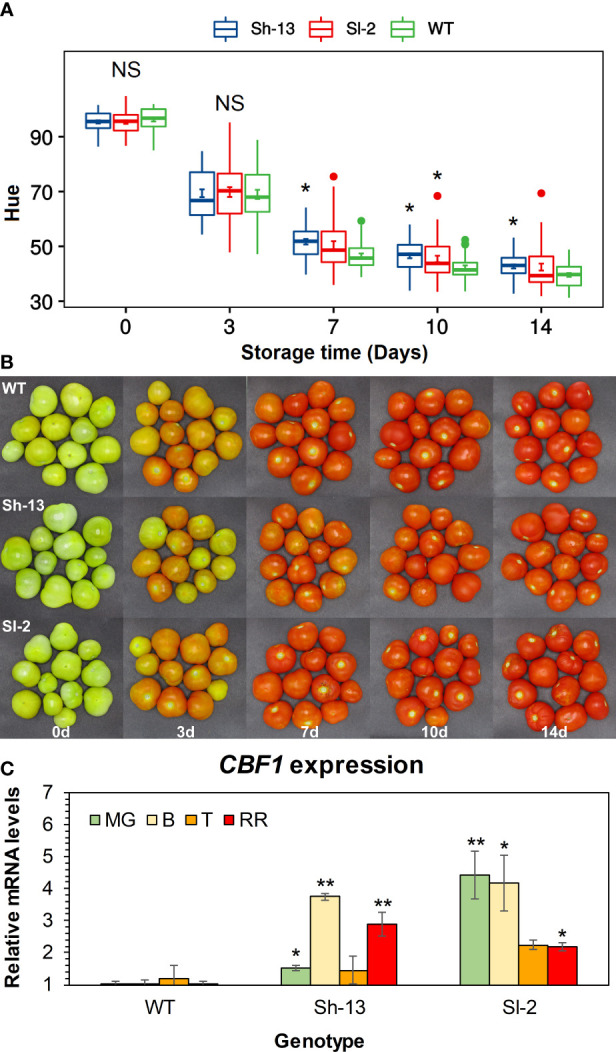
Characterization of *CBF1*-overexpression lines (Sh-13 and Sl-2) and WT fruit. **(A)** Hue angle of breaker fruit stored at 20°C for up to 14 days. Boxes with asterisks are significantly different (*p*< 0.05) than WT fruit by Dunnett’s test. Error bars are included. **(B)** External appearance of breaker fruit stored at 20°C for up to 14 days. **(C)** *CBF1* expression of fruit harvested at different maturity stages. Transcript accumulation was determined by quantifying both native and transgenic expression of this gene at the mature green (MG), breaker **(B)**, turning (T) and red ripe (RR) stages. WT fruit at each maturity stage were used as the calibrator. Columns with asterisks are significantly different (*p*< 0.05) to WT fruit at each stage by unpaired *t*-test. NS, Not significant.

Fruit morphology was altered in some of the transgenic lines (**Supplementary Table S2**). External color in freshly harvested fruit was similar in Sh-13, Sl-2, and WT fruit (**Figure 3**). Color differences between transgenic and non-transgenic lines at 20°C became significant after one week of storage and were maintained until the end of the experiment. As evidenced by higher Hue values, transgenic genotypes were unable to ripen to the same extent as WT fruit (**Figure 3**).

The expression of *CBF1* was characterized in fruit harvested at different stages of maturity, and in cold-exposed seedlings by RT-qPCR. In fruit, relative to the wild-type, all maturity stages had higher *CBF1* expression, except for Turning fruit (**Figure 3C**). Seedlings from the WT and transgenic genotypes showed *CBF1* upregulation after 12 or 24 hours of storage at 0°C, compared to samples kept at room temperature. In WT seedlings, *CBF1* expression peaked after 24 h, showing a 936.4-fold increase, while Sh-13 and Sl-2 reached 41.4- and 15.2-fold increase, respectively (**Supplementary Figure S1**).

### 3.2 Cold storage induced transgene upregulation in fruit

**Figure 4:**
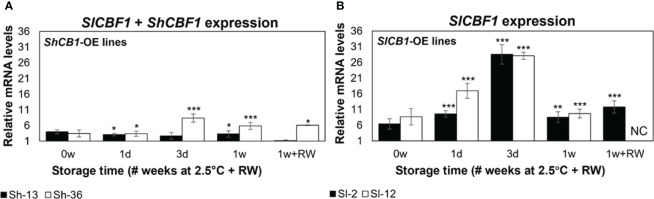
Relative *CBF1* expression of transgenic fruit. Columns are the mean ± SE of breaker fruit stored at 2.5°C for up to 1 week, or followed by transfer to 20°C for 3 days (‘1w+RW’). **(A)** Expression of *SlCBF1* and *ShCBF1* in *ShCBF1*-OE lines. Due to*SlCBF1* and *ShCBF1* sequency similarity, total *CBF1* expression was measured by designing primers to a conserved region between both genes. **(B)** *SlCBF1* expression in *SlCBF1*-OE lines. Transcript accumulation was determined by quantifying both native and transgenic expression of this gene. ‘NC’ not calculated. WT fruit at each time point were used as the calibrator. Columns with asterisks are significantly different (*p*< 0.05) to the WT fruit at each time point by unpaired *t*-test.

Expression of *CBF1* was assessed in two ways. First, CBF1 transcripts were monitored during fruit storage to determine potential changes over time (**Supplementary Figure S2**). Secondly, CBF1 transcripts were monitored at each timepoint in the engineered fruit and compared to that in WT, to determine upregulation due to the transgene. (**Figure 4**). Due to *ShCBF1* and *SlCBF1* sequence similarity, ShCBF1 transcript accumulation was determined by measuring the total *CBF* expression in lines Sh-13 and Sh-36. In Sh-13 and Sh-36, total *CBF1* expression was stable over storage (**Supplementary Figures S2C**, **S2E**). Relative to WT fruit, transcripts were upregulated at all time points in Sh-36 (**Figure 4A**).

*SlCBF1*-OE lines (Sl-2 and Sl-12) were developed to increase the number of transcripts of the native *CBF1* gene. In these lines, transcript abundance decreased after one week of storage (**Supplementary Figures S2D**, **S2F**). Relative to freshly harvested fruit, *CBF1* expression was upregulated at all time points in both lines, increasing from day 1, peaking at day 3, and declining after one week of cold exposure (**Figure 4B**).

In the *ShCBF1*-OE (**Figure 4A**) and *SlCBF1*-OE lines (**Figure 4B**), transcript accumulation did not change at 0w compared to the WT. Expression of *SlCBF1* in the WT did not vary after one day of storage (**Supplementary Figures S2A**, **S2B**).

### 3.3 Deviation in color development between *CBF1*-OE lines and WT fruit

**Figure 5:**
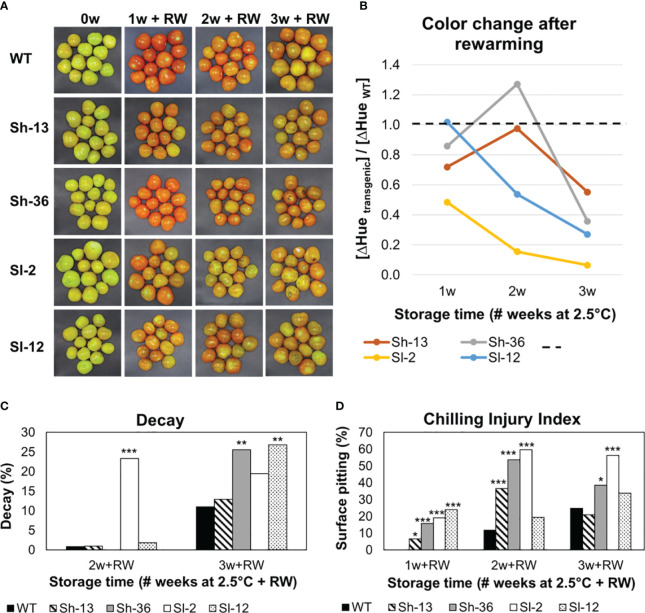
External appearance of cold-stored WT and transgenic tomato fruit. Breaker fruit were stored at 2.5°C for up to 3 weeks. After 1, 2 or 3 weeks of storage, fruit were rewarmed (RW). **(A)** Visual color changes. **(B)** Effect of RW on objective color change: the ratio of ΔHue in the transgenic *vs*. WT lines. ΔHue was calculated as the difference between the average of 1, 2 or 3 weeks of cold storage, and the same time followed by RW. WT fruit is represented by the dashed line. Values below 1 indicate that fruit did not redden to the same extent as the WT after rewarming. In contrast, fruit that turned as red or redder than the WT will present values equal to or greater than 1, respectively. **(C)** Decay %. **(D)** Chilling injury index. Scores were determined after RW. Columns with asterisks are significantly different (*p*< 0.05) to WT fruit by unpaired *t*-test.

Fruit from wild-type and transgenic lines presented different objective color values during cold storage and after rewarming (**Figure 5**).

Cold storage suppressed the green-to-red transition in all fruit (**Figure 5A**). Rewarming normally elicits fruit-reddening, but remarkably, reddening during rewarming was attenuated in the transgenic lines relative to the wild-type fruit as shown in **Figure 5B**.

### 3.4 Development of PCI symptoms was aggravated in transgenic fruit

Fruit were cold-stored and transferred to 20°C for three additional days to induce PCI. The presence of surface lesions or pitting (Chilling Injury Index, CII) and deterioration in the form of decay, were recorded. Wild-type fruit consistently had the lowest CII scores among all genotypes, maintaining levels below 30% after 2 or 3 weeks of cold storage, and rewarming (**Figure 5A**). In contrast, transgenic fruit developed pits after just one week at 2.5°C, especially in Sh-36 and Sl-2. There was a slight decrease in CII between weeks 2 and 3, which was linked to the pitted lesions becoming ‘swollen’ in appearance (**Supplementary Figure S3G**).

The highest incidence of decay was observed in Sh-36, Sl-2, and Sl-12 (**Figure 5B**). A myriad of symptoms was recorded, i.e., severe discoloration, wrinkles around the stem scar, and surface ‘translucency’ (**Supplementary Figure S3**), but they were absent or minimized in the WT.

Degradation of RNA samples obtained from the transgenic fruit was observed and verified during agarose gel electrophoresis (data not shown) and may be connected to the phenotypic deterioration induced by *CBF1* overexpression.

**Figure 6:**
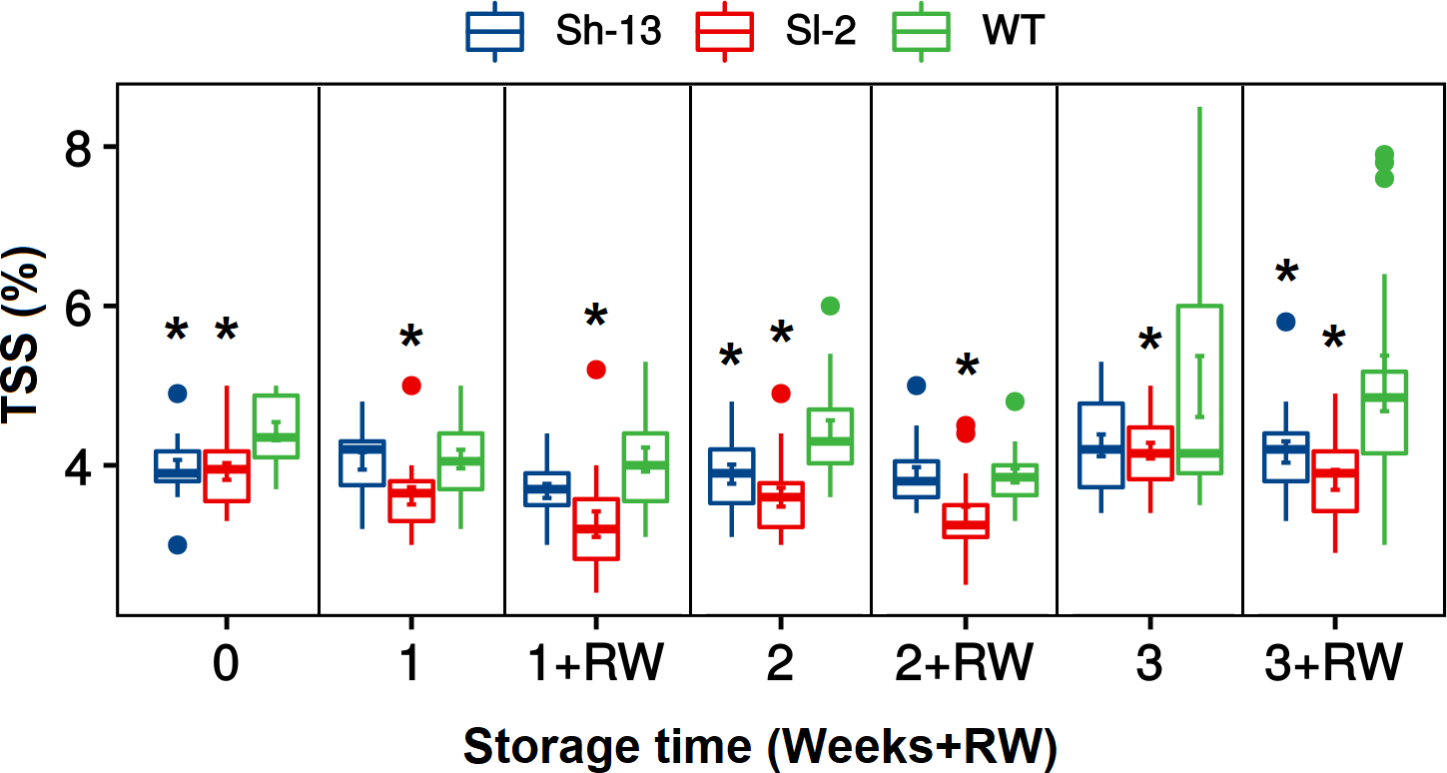
TSS changes of *CBF1*-overexpression lines (Sh-13 and Sl-2) and WT fruit. Breaker fruit were stored at 2.5°C for 7, 14 or 21 days, or rewarmed for three days. Boxes with asterisks are significantly different (*p*< 0.05) than WT fruit at the same time point by Dunnett’s test.

Based on their contrasting PCI phenotype, further analyses were conducted on Sl-2 and Sh-13 fruit in addition to the WT. The total soluble solids (TSS) of the transgenic fruit was consistently lower than non-transgenic samples throughout cold storage, as well as after rewarming (**Figure 6**).

**Figure 7:**
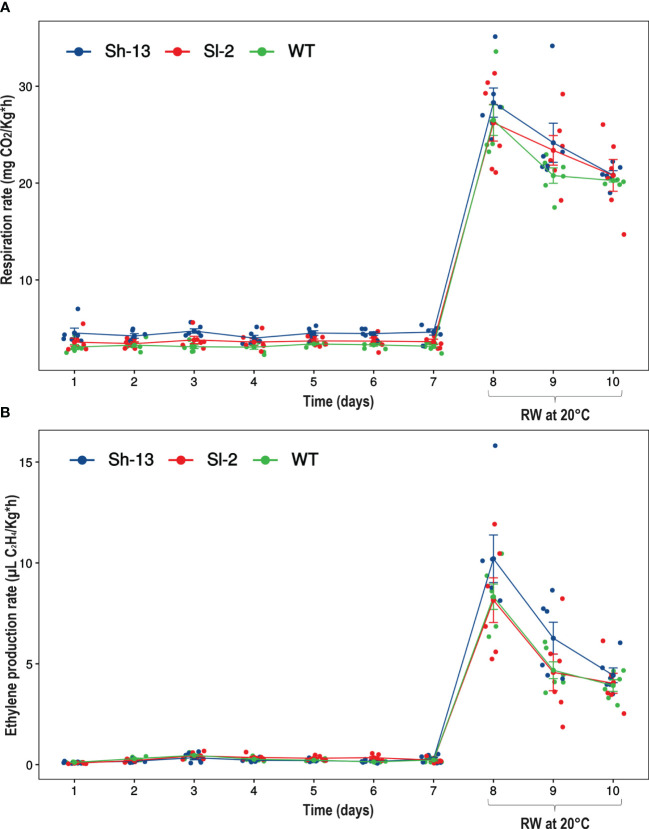
Gas production rates of *CBF1*-overexpression lines (Sh-13 and Sl-2) and WT fruit. **(A)** Respiration rate. **(B)** Ethylene production rate. Breaker fruit were stored at 2.5°C for up to 7 days, and then rewarmed for three days. Each point represents the mean ± SE.

Respiration rates were significantly higher in Sh-13 compared to non-transgenic fruit throughout cold storage, except for day 4 (**Figure 7A**). However, after rewarming all three genotypes were the same. Interestingly, CO_2_ emission in Sh-13 only increased 512.8% which was lower than in Sl-2 (623.5%) and WT (744%) fruit.

Ethylene emission rates were similar between transgenic lines and the WT, with changes on days 2 and 6. After transfer to 20°C, values increased ~3,500% (**Figure 7B**).

### 3.5 Volatile compounds

**Figure 8:**
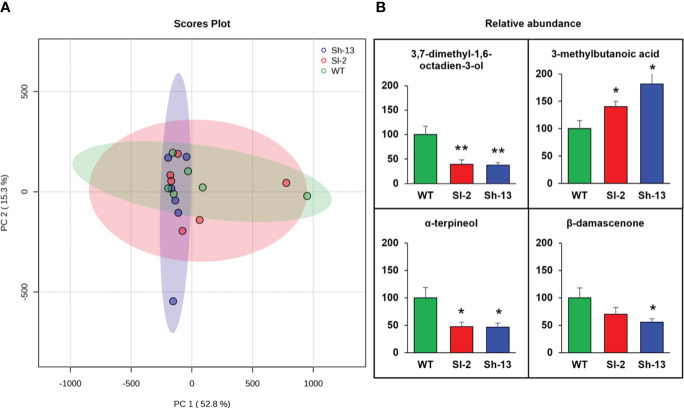
Volatile compounds assayed in the *CBF1*-overexpression lines (Sh-13 and Sl-2) and WT fruit. Breaker fruit were stored at 2.5°C for 14 days, and then rewarmed for three days. **(A)** PCA generated from SPME/GC-MS analysis of volatile compounds. **(B)** Relative abundance of volatile compounds with statistical significance across genotypes. For each compound, average levels of WT were arbitrarily set to 100 for comparison. Bars are the mean ± SE of fruit. Columns with asterisks are significantly different (*p*< 0.05) than WT fruit by *t*-test.

The level of volatile organic compounds (VOCs) in Sh-13, Sl-2, and WT fruit chilled at 2.5°C for 2 weeks followed by RW was determined by solid phase microextraction (SPME) coupled with gas chromatography–mass spectrometry (GC-MS). A total of 34 VOCs were identified from the fruit headspace (**Supplementary Table S6**; **Supplementary Figure S7**). Principal component analysis (PCA) revealed 52.8% and 15.3% of the variance was explained by the first and second principal components, respectively. Samples from the transgenic fruit did not separate from those of the WT (**Figure 8A**). However, transgenic fruit had a lower relative abundance of the monoterpenes α- terpineol, 3,7-dimethyl-1,6-octadien-3-ol, and β- damascenone, higher levels of the fatty acid 3-methylbutanoic acid (**Figure 8B**).

### 3.6 Transcriptomic analysis by RNA-Seq

Differentially expressed genes (DEGs) in the Sh-13, Sl-2 transgenic fruit *vs*. the WT after storage at 2.5°C for either 6 hours or 1 week were identified. The data from the WT fruit were compared against the pooled data of the transgenics.

**Figure 9:**
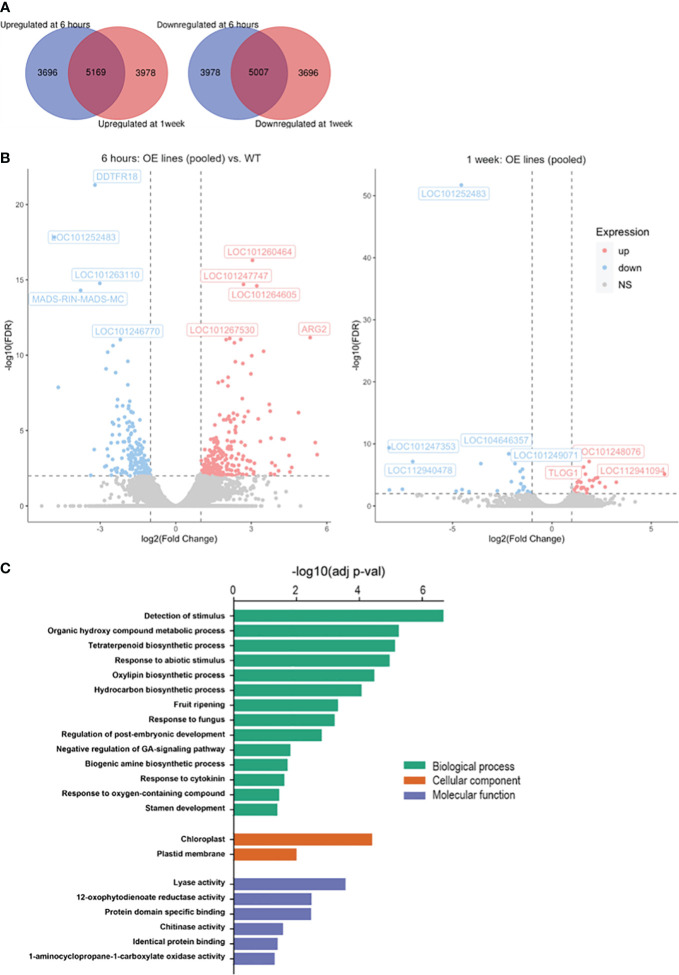
RNA-Seq data of *CBF1*-overexpression lines (Sh-13 and Sl-2) and WT fruit. Breaker fruit were stored at 2.5°C for 6 hours or 1 week. **(A)** Venn diagrams representing the up- (left) or downregulated (right) genes of pooled Sh-13 and Sl-2 samples in relation to WT fruit. **(B)** Volcano plot representing the highest up- or downregulated genes in pooled transgenic samples (OE lines) compared to WT fruit after 6 h (left) or 1 w (right) of cold storage. FDR: False discovery rate; NS: non-significant. FDR< 0.01, fold change > 2. **(C)** Gene ontology enrichment analysis indicating biological processes (BP), cellular component (CC), and molecular function of differentially expressed genes (DEGs) in pooled transgenic samples compared to WT fruit. Adjusted *p*-value<0.05. Analysis was carried out using the g:Profiler online tool, and *S. lycopersicum* as a reference organism.

After 6 hours and 1 week of cold storage, 5,169 genes were induced collectively in the transgenics, whereas 5,007 genes were repressed (**Figure 9A**). In Sh-13 and Sl-2 fruit, a range of 5,832-7,141 DEGs were shared by both genotypes in the cold (**Supplementary Figure S8**). The similarity of these results strengthened the justification for treating the combined results from the *CBF1*-OE lines as biological replicates of the same genotype. Among the greatest upregulated annotated genes were *ARGINASE2* (*ARG2*), aquaporins *PIP1-7* and *PIP2-1*, protein *LURP-one*-*related 14-like*, and *Cytokinin riboside 5’-monophosphate phosphoribohydrolase LONELY GUY 1* (*TLOG1*). As for the downregulated genes, *AT-hook motif nuclear-localized protein 17* (*AHL17*), *RIPENING INHIBITOR* (*RIN*), Ripening regulated protein *DDTFR18*, *Acyl-CoA-binding domain-containing protein 3* (*ACBD3*), *Vestitone reductase-like*, *Expansin-like B1* (*EXLB1*) and a chloroplastic Protein *CutA* were identified (**Figure 9B**; **Supplementary Table S7**).

To identify the most relevant gene groups affected by *CBF* overexpression in cold stored fruit, gene ontology (GO) enrichment analysis was conducted. Some of the biological processes altered include the detection and response to abiotic stimulus and fungus, and fruit ripening. Among the cellular compartments involved were the chloroplast and plastid membrane, whereas lyase and chitinase activities, and the ethylene biosynthesis pathway were shown as modulated molecular functions in *CBF1*-OE fruit compared to the WT (**Figure 9C**).

### 3.7 Fruit chilling affected wild-type seed and seedling features

To develop a more holistic and integrative view of chilling injury in the context of a continuum between fruit and progeny responses to cold stress, we explored if fruit cold response would influence the germination of tomato seeds derived from those fruit. This information would allow us to establish a baseline to assess the vegetative cold tolerance of the *CBF1*-OE lines.

**Figure 10:**
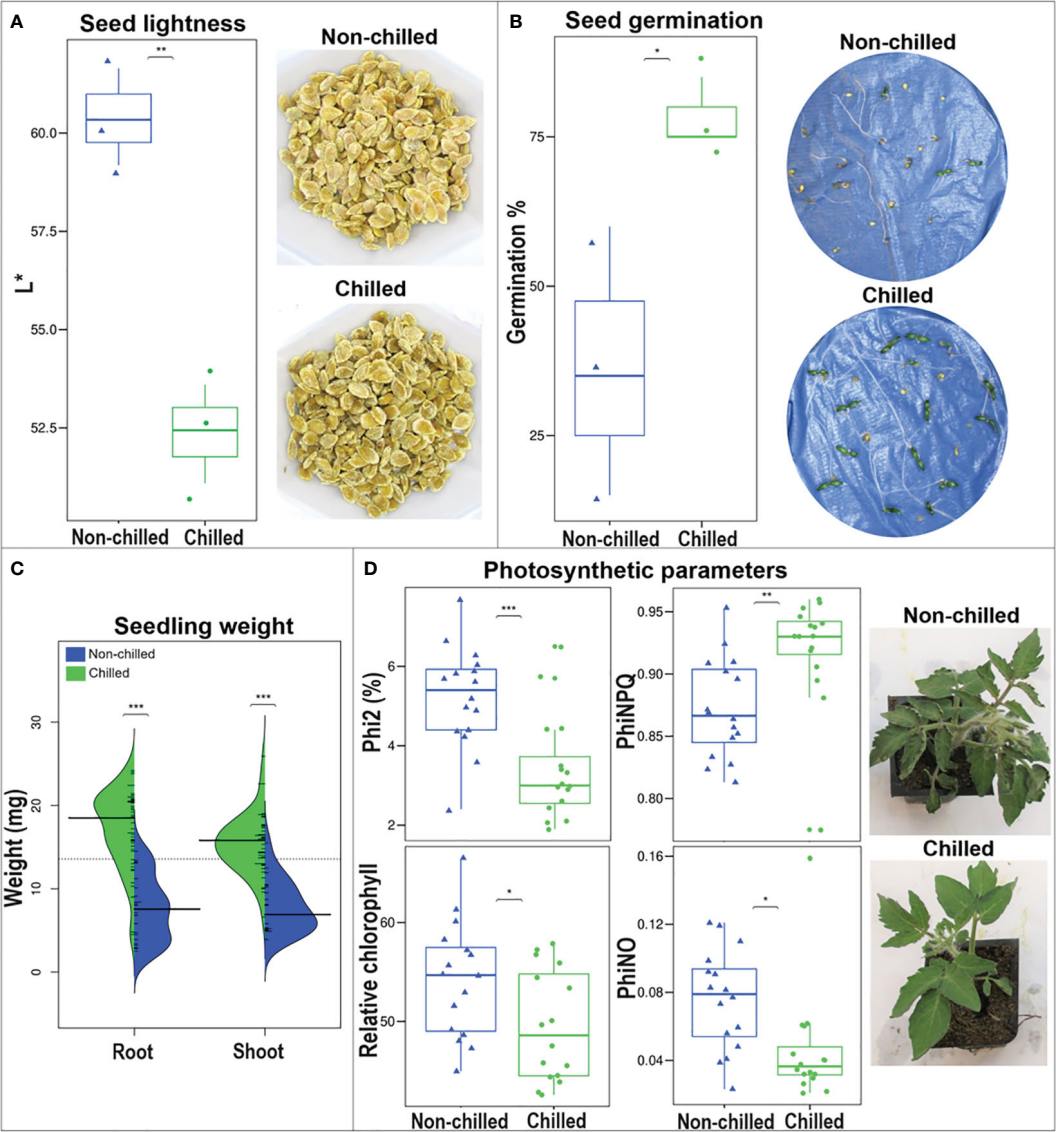
Seed and seedling physiological parameters of WT tomato. Seeds were collected from ‘non-chilled’ and ‘chilled’ fruit. **(A)** L* value box plot and seed appearance. **(B)** Germination percentage box plot after 7 d at 20°C. **(C)** Shoot and root fresh weight violin plot after 1 week at 20°C and seedling appearance after 5 days at 20°C. **(D)** Photosynthetic performance and appearance of plants after 72 hours at 0°C. Asterisks indicate significant difference (*p*< 0.05) by unpaired *t*-test.

WT seeds were collected from wild-type fruit at breaker stage, induced to PCI (‘chilled’), or kept at room temperature (‘non-chilled’). The L* value, assesses sample brightness and was lower (darker) in chilled compared to non-chilled seeds (**Figure 10A**). Seed weight was higher (*p* > 0.05) in chilled (3.9 ± 0.2 mg/seed) compared to non-chilled (2.8 ± 0.4 mg/seed) seeds.

A higher percentage of chilled seeds germinated (78.3%) compared to the non-chilled samples (36.7%) (**Figure 10B**), and the fresh mass of ‘chilled’ hypocotyls (shoot) and roots was also superior after 7 days of germination at room temperature (**Figure 10C**).

### 3.8 Photosynthetic performance of wild-type seedlings was affected by cold stress

Seedlings at the six-leaf stage, obtained from WT chilled and non-chilled fruit, were stored at 2.5 or 0°C for up to 72 hours, and their photosynthetic performance was monitored every 24 hours on the fourth leaf. No differences were detected between the 2.5°C and control seedlings (**Supplementary Table S3**), however, photosynthetic responses differed after storage at 0°C, and particularly after 72 hours (**Figure 10D**; **Supplementary Table S4**). Responses to cold were heterogeneous among parameters. Some were lower in chilled, compared to non-chilled seedlings: 1) quantum yield of PSII, or Phi2, which is the percentage of incoming light directed into PSII; 2) Linear Electron Flow (LEF) or the flow of electrons from antennae complexes into PSII; 3) PhiNO, a ratio of processes that inhibit photosynthesis, when absorbed light is not dissipated by non-photochemical quenching (Kuhlgert et al., 2016).

Two parameters related to Non-Photochemical Quenching (NPQ) were also recorded i.e., NPQt and PhiNPQ (**Supplementary Table S4**; **Figure 10D**). They denote the ratio of incoming light in terms of excess energy that is dissipated as heat (Müller et al., 2001). These two variables progressively increased in both ‘chilled’ and ‘non-chilled’ samples over the course of the experiment, however, after 72 hours, they were higher in ‘chilled’ compared to ‘non-chilled’ seedlings.

The relative chlorophyll content of seedlings was consistently higher in ‘non-chilled’ compared to ‘chilled’ seedlings after 24, 48, and 72 hours at 0°C, matching qualitative observations (**Figure 10D**). However, at 2.5°C, values did not differ (**Supplementary Table S3**). Overall, visual attributes of non-chilled seedlings were more affected by cold storage after three days than chilled samples. Wilting and leaf curling was accentuated in non-chilled seedlings.

### 3.9 Transgenic *CBF1*-OE lines had similar photosynthetic performance during cold storage

Of the engineered genotypes studied, Sl-2 and Sh-13 fruit had the highest and lowest incidence of PCI symptoms, respectively (**Figure 5**), and Sl-12 showed more aberrant vegetative growth compared to Sh-13 (**Supplementary Figure S4**). Therefore, Sl-2 and Sh-13 were selected to characterize their photosynthetic performance during cold storage at the seedling stage. The aim of this test was to determine if fruit and seedling response to cold stress were correlated, and it was instructive to choose lines with distinct PCI responses.

Seedlings from Sh-13 and Sl-2 lines at the 6^th^-leaf stage, obtained from non-chilled fruit, were exposed to 0°C for up to 72 hours. Photosynthetic parameters were monitored every 24 hours on the fourth leaf. LEF progressively decreased over time in both lines and reached the lowest level on day three, with Sl-2 being higher than Sh-13 (**Supplementary Table S5**). The rest of the parameters were similar between genotypes at all time points, but indicators of non-photochemical quenching were higher in Sh-13 (*p* > 0.05). Phenotypically, Sl-2, which showed the highest severity PCI symptoms in the fruit, had a greater incidence of leaf curling and wilting after cold-storage than Sh-13 (**Supplementary Figure S5**).

Absolute parallels between the responses of wild-type and transgenic seedlings cannot be established, due to differences in light intensity between experiments.

## 4 Discussion

### 4.1 The use of the *RD29A* promoter did not ameliorate pleiotropic effects due to *CBF1* overexpression

Constitutive overexpression of *AtCBF1* (Hsieh et al., 2002a, 2002), *AtCBF3* (Zhang et al., 2004) or *SlCBF1* (Zhang et al., 2004) in tomato increased abiotic stress tolerance, but at the cost of growth and development. The use of stress-inducible promoters yielded variable results. In *ABRC1*::*AtCBF1* and *RD29A*::*AtCBF1* plants, developmental abnormalities were absent, but it is unclear if growth chambers or greenhouse were used in these studies (Lee et al., 2003; Singh et al., 2011). Growth chamber-grown *RD29A*::*AtCBF3* plants had a phenotype consistent with *CBF* overexpression (Hu et al., 2015), whereas dexamethasone-inducible lines overexpressing *AtCBF1* did not manifest abnormalities during vegetative growth (Albornoz et al., 2023). In this study, we used *RD29A* as a cold-inducible promoter to preferentially overexpress *ShCBF1* or *SlCBF1* in harvested fruit under chilling, to minimize plant pleiotropic effects associated with constitutive overexpression. The *RD29A* promoter has been extensively studied in Arabidopsis and widely used in ectopic expression systems, including tomato (Singh et al., 2011), potato (Pino et al., 2007) and tobacco (Qiu et al., 2012). In this study, *RD29A* was selected as a promoter based on its responsiveness to cold stress, and the minimized leakiness reported by other authors.

Our plants were phenotypically abnormal, similar to transgenic lines constitutively expressing *CBF1* (Hu et al., 2015). Therefore, our greenhouse led to variable, ‘stressful’ conditions, that activated *RD29A* and the transcription of *CBF1* in the absence of an imposed, designed stress, i.e., cold. The *RD29A* promoter contains *cis*-elements that are responsive to various abiotic stresses involving changes in osmotic potential, low temperature, dehydration, salinity, and ABA application (Yamaguchi-Shinozaki and Shinozaki, 1994). In soybean, the activity of the transgenic *RD29A* promoter showed a strong induction with low soil moisture; however, under optimally-watered conditions, its activity decreased but it was not completely suppressed (Bihmidine et al., 2013). In petunia (Estrada-Melo et al., 2015), tobacco (Kasuga et al., 2004), Arabidopsis (Chen et al., 2010), and potato (Huynh et al., 2014), leaky induction of this promoter was also documented.

Leaky induction of *CBF1* could be associated with changes at the whole plant level, unrelated to the phenomenon under study, i.e., cold stress response in fruit. Nevertheless, the postharvest responses of cold-stored fruit from the transgenic lines and independent transformation events confirmed that the phenotype was consistent across genotypes.

### 4.2 *CBF1* overexpression aggravated PCI symptoms and altered VOCs in transgenic fruit

The CBF regulon in tomato plants constitutively overexpressing *AtCBF3* or *SlCBF1* (formerly *LeCBF1*), consisted of two dehydrins (with key roles in cryoprotection), and one putative proteinase inhibitor (Zhang et al., 2004). A higher synthesis of protective molecules may explain increases in cold tolerance due to *CBF* constitutive overexpression (Jaglo-Ottosen et al., 1998; Hsieh et al., 2002a).

We hypothesized that the induction of additional *CBF1* transcripts in tomato fruit during postharvest chilling, would increase cold tolerance and ameliorate PCI symptoms due to increased synthesis of transcripts associated with protective proteins. Furthermore, the regulated overexpression of *CBF1* would circumvent pleiotropic effects during plant growth and development.

We were able to induce *CBF1* overexpression in fruit postharvest chilling, consistent with previous reports (Albornoz et al., 2023), thus validating the use of this regulated-expression approach. Expression of *CBF1* peaked after three days of cold exposure relative to wild-type fruit: 8-fold in *ShCBF1*-OE, and 28-fold higher in *SlCBF1*-OE lines.

Remarkably, *CBF1* overexpression aggravated PCI symptoms. Fruit were unable to complete the ripening process, and deterioration was accelerated when compared to wild-type fruit under the same conditions. Lines Sl-2, Sl-12 and Sh-36 had the highest incidence of pitting and decay, which correlated with *CBF1* overexpression data. These observations corroborate the connection between *CBF1* upregulation and worsening of PCI symptoms. Expression and phenotypic differences between transformation events of the same transgenic construct may be due to position effects (van Leeuwen et al., 2001), somaclonal variation due to chromosomal rearrangements during tissue culture (Manchanda et al., 2024), or transgene copy number variations. Insertion of multiple copies of a transgene could lead to silencing and hinder the interpretation of progeny segregation ratios (Collier et al., 2017). Previous reports altering the expression of CBFs in tomato fruit have revealed mixed results. The absence of a severe phenotype during fruit postharvest chilling of *AtCBF1*-overexpression lines may be due to the limited storage length used (Albornoz et al., 2023). On the other hand, fruit from *slcbf1* mutants showed aggravated PCI symptoms after rewarming, compared to the wild-type (Yu et al., 2024).

Fruit decay susceptibility is commonly associated with pathogen accessibility to cell wall components, which is facilitated by ripening (Cantu et al., 2008). However, *CBF1* transgenic fruit were ripening-impaired, yet had a higher incidence of decay. It is tempting to hypothesize that *CBF1* regulates cell wall degradation via a ripening-independent pathway.

Higher decay and deterioration of transgenic fruit mirrored our volatile data. *CBF1*-overexpression fruit had a higher relative abundance of 3-methylbutanoic acid, which is associated with an unpleasant ‘cheesy’, ‘sweaty’ aroma in tomato pomace samples fermented by yeast, compared to unfermented samples (Bartoshuk and Klee, 2013; Güneser et al., 2015). Other compounds were less abundant in the transgenics than in WT fruit. α-terpineol is a defense VOC over-emitted in tomato plants that resisted a bacterial disease (López-Gresa et al., 2017), and it is perceived as a pine-like, woody aroma, valued in the flavors and fragrances industry (Sales et al., 2020). This correlates with the lower decay incidence observed in WT fruit. Synthesis of β-damascenone and 3,7-dimethyl-1,6-octadien-3-ol (linalool) was greater in ripe fruit (Mir et al., 2004; Rambla et al., 2014) and it is associated with the lower color development in our chilled transgenic samples compared to the WT.

The CBF family of transcription factors integrate environmental and hormonal signals, to promote plant survival under stresses (Kurepin et al., 2013). There is evidence of an interaction between ethylene biosynthesis and *SlCBF1* expression in response to postharvest chilling stress, and, during ripening (Albornoz et al., 2019). Exogenous application of ethylene in addition to cold storage, led to *SlCBF1* upregulation (Zhao et al., 2009b). Further, the suppression of ethylene biosynthesis was linked to lower *SlCBF1* transcript accumulation and an increase in chilling sensitivity in tomato fruit (Yu et al., 2019). Our results are consistent with an increase in fruit susceptibility to chilling due to *CBF1* upregulation, but they did not correlate with ethylene biosynthetic rates.

CBFs repress the cellular content of the hormone gibberellin (GA), and this may lead to pleiotropic effects seen in *CBF1* transgenic lines. Lower GA levels have been shown to underscore the dwarfism observed in transgenic lines overexpressing *CBF* cloned from Arabidopsis (Achard et al., 2008; Zhou et al., 2017). This phenotype has also been observed in potato (Pino et al., 2008), rice (Lee et al., 2004; Moon et al., 2019), apple (Wisniewski et al., 2011), and in our transgenic plants. When we treated *CBF1*-OE seedlings with 5 µL L^-1^ GA they grew taller than water-treated samples but not to WT levels (results not shown). GAs may also influence fruit ripening, although the mechanism is not yet clear (Park and Malka, 2022). Mature green Micro-Tom fruit treated with exogenous GA ripened slower than the non-treated fruit (Li et al., 2019). In our lines, fruit GA levels were not assessed, however, fruit sensitivity and responsivity to altered GA may differ, causing differential responses in fruit ripening.

Based on our results, we propose that CBFs are involved in critical processes in plant growth and development. The higher rate of fruit deterioration and susceptibility to postharvest decay suggest CBFs are important regulators in fruit ripening and senescence under stress conditions. Further research should address its interplay with plant hormones and other key players in the cold stress response pathway.

### 4.3 Transcriptomic changes in *CBF1*-OE lines were consistent with phenotypic data

Lines Sh-13 (*ShCBF1*-OE) and Sl-2 (*SlCBF1*-OE) were selected for RNASeq analysis after short- (6 h) or long-(1 w) term cold storage as they developed similar PCI symptoms.

Upregulation of genes involved in abiotic and biotic stress response and hormone biosynthesis was detected after cold storage. *ARGINASE2* (*ARG2*) is involved in arginine metabolism and has been linked to methyl jasmonate-induced defense responses to *Botrytis cinerea* (Min et al., 2020), and PCI tolerance in heat-shock exposed tomato fruit (Zhang et al., 2013). Modulation of aquaporin expression has been seen in tomato plants exposed to abiotic stresses, including salt, drought, and elevated carbon dioxide (Fang et al., 2019; Jia et al., 2020). The *LURP1* gene is involved in biotic stress responses in Arabidopsis (Knoth and Eulgem, 2008; Bektas et al., 2016). Interestingly, *TLOG1* codes for a cytokinin-activating enzyme associated with tuber formation in tomato plants overexpressing this gene (Eviatar-Ribak et al., 2013) and suggests that hormone homeostasis was compromised in our transgenic lines.

Other genes related to biotic and abiotic stress response, and developmental processes, were downregulated in *CBF1*-OE fruit in response to cold. For instance, *AHL* genes are positive regulators of cold tolerance in trifoliate orange (Dahro et al., 2022). *Acyl-CoA-binding domain-containing proteins* (*ACBP*s) and *EXLB1* are involved in stress responses and ripening in tomato (Chatterjee et al., 2007), Arabidopsis (Xue et al., 2014), and apple (Chen et al., 2022). Vestitone reductase-like proteins participate in the biosynthesis of the defense compounds phytoalexins (Jeandet et al., 2013). Repression of these genes is consistent with the higher chilling sensitivity and impaired stress response presented by our transgenic fruit.

Downregulation of the transcription factor and master ripening regulator, *RIN*, (Li et al., 2020) can partly explain the inability of *CBF1*-OE fruit to resume ripening to WT levels after cold storage, and its early repression (6 h) suggests it may be a candidate for predicting PCI onset. Consistent with this, *DDTFR18*, with putative roles in ripening and ethylene response (Giovannoni et al., 1999) was also repressed. In WT Micro-Tom fruit, *DDTFR18* was cold-induced (Weiss and Egea-Cortines, 2009), but ectopic *CBF1* overexpression may have altered this response.

### 4.4 Accelerated fruit deterioration due to *CBF1* overexpression may be advantageous for seed dispersal

Ripening is an early stage in the continuous process leading to senescence (Oeller et al., 1991; Klee and Giovannoni, 2011), and it may include changes in fruit color, firmness, and flavor. PCI compromises the normal transition between chloroplast and chromoplast during ripening (Gomez et al., 2009), even after transfer to warmer conditions (Albornoz et al., 2019). However, in the current study, *CBF1* overexpression not only accentuated the inability of fruit to resume ripening after rewarming that was already observed in WT fruit, but it also increased susceptibility to decay, thus triggering accelerated deterioration and tissue disassembly.

**Figure 11:**
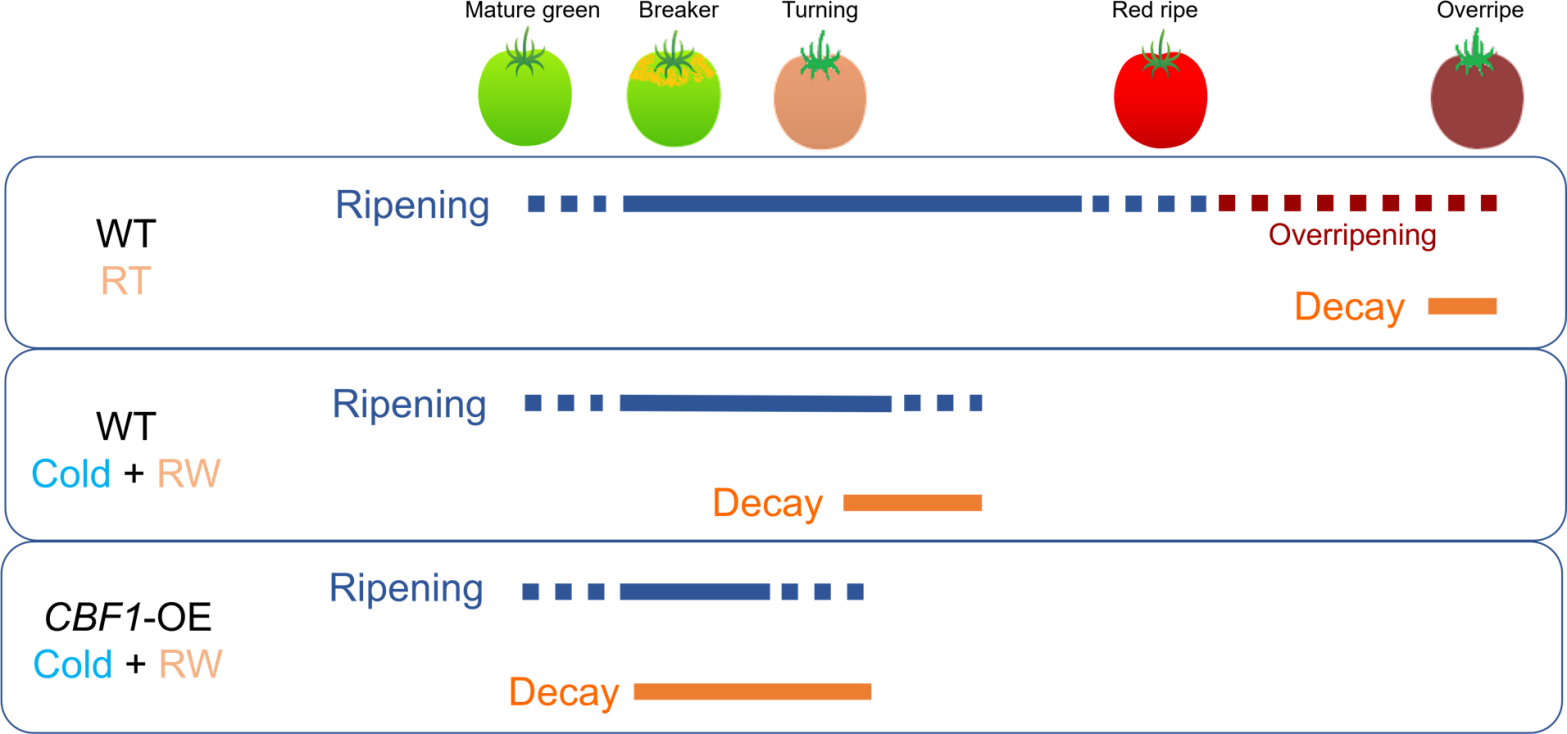
Schematic representation of ripening and decay processes in postharvest fruit at different stages. Shown are wild-type (‘WT’) and *CBF1*-overexpression (‘*CBF1*-OE’) lines. RT, room temperature; RW, rewarming of fruit at 20°C. The initiation and period over which decay occurs vary due to genotype, maturity stage at harvest, and environment.

Fruits evolved as vehicles for seed dispersal (Tanksley, 2004; Knapp and Litt, 2013). During ripening, a complex and dynamic set of changes that modify fruit appearance (Khudairi, 1972), firmness (Shackel et al., 1991), aroma (Zou et al., 2018) and flavor (Klee and Giovannoni, 2011) take place (Giovannoni, 2004). This reconfiguration makes the fruit more attractive for seed-dispersing animals, including humans, and is known as the dispersal syndrome (Herrera, 2002). As ripening progresses, fruit becomes more susceptible to pathogen infestation (Gapper et al., 2013; Alkan and Fortes, 2015). Since color development is terminated early in *CBF1*-overexpression lines, we propose that the aggravation of surface pitting, decay, and other symptoms of global fruit disassembly, might constitute a strategy to facilitate seed dispersal in absence of other traits normally elicited by ripening. Understanding the basis of fruit deterioration caused by PCI might help to develop solutions for maintaining fruit quality during refrigeration (**Figure 11**).

### 4.5 Chilling in wild-type fruit improved progeny fitness under control and cold stress conditions

To explore a potentially advantageous role of PCI in the next plant generation, we collected seeds from WT fruit that were allowed to ripen at room temperature (‘non-chilled’), and from cold-stored fruit followed by RW (‘chilled’). Seed color and weight, seedling germination percentages and fresh weight were recorded at room temperature.

Cold storage caused seed browning and discoloration, triggered by cell decompartmentalization (Boonsiri et al., 2007; Massolo et al., 2011; Albornoz et al., 2019). The higher weight (*p* > 0.05) in chilled seeds could be associated with greater sugar translocation into the seeds during rewarming due to enhanced fruit respiration (Kitano et al., 1998). We observed similar enhanced respiration in fruit rewarmed after 2.5°C-storage compared to fruit at 12.5°C (Albornoz et al., 2019). Thus, chilled seeds could also have experienced higher sugar translocation from the fruit after rewarming.

Stratification is the process of disrupting seed dormancy by exposure to moist chilling conditions (Bentsink and Koornneef, 2008). Treatments like thermal hardening (Nawaz et al., 2009; Yari et al., 2012), can support our observations of enhanced germination percentage and seedling weight in samples from chilled fruit. These treatments alter hormonal signaling and also interact with mechanisms crucial to the perception of environmental cues (Bentsink and Koornneef, 2008).

In the presence of light, chilling stress in cold-sensitive species causes the desynchronization of antenna complexes and PSII (Garstka et al., 2007), causing photoinhibition and damage (Garstka et al., 2007; Knight and Knight, 2012; Onaga and Wydra, 2016). To better understand how cold acclimation of the seeds *in fructa* affects the cold tolerance of the resultant seedlings, we exposed WT ‘chilled’ and ‘non-chilled’ seedlings to 0°C for three days and compared their photosynthetic responses. We observed that seed chilling tolerance was enhanced when contained in a maternal organ (fruit), thus suggesting that an adaptive mechanism to cope with cold stress was transmitted across generations from the maternal tissue to the embryo. Evidence of chilling acclimation exists, but with the primary stress occurring on seedlings, not fruit-contained seeds (Zhou et al., 2012; Barrero-Gil et al., 2016). Photosynthetic results suggest 1) a reduction of the flow of energy and electrons directed towards PSII, as well as the proportion of photosynthetic inhibition due to excess energy, and relative chlorophyll content, and 2) an increase in mechanisms associated with non-photochemical quenching to minimize overexcitation. Chilling-acclimated seedlings may have reduced the amount of light used in photosynthesis to minimize damage, and reducing total chlorophyll is an adaptive mechanism to lower absorbed light (Barrero-Gil et al., 2016).

### 4.6 Transgenic fruit and seedling responses to chilling stress highlight the heterogeneity of CBF1 roles in different developmental stages

To gain insight into the response of *CBF1*-OE lines to cold stress during early development, seedlings from lines Sh-13 and Sl-2 were exposed to 0°C for three days. A trend towards higher light absorption (*p <* 0.05) and lower non-photochemical quenching (*p* > 0.05) was observed in Sl-2 compared to Sh-13. This revealed that Sl-2 experienced more photoinhibition and excitation pressure than Sh-13, suggesting that high levels of *SlCBF1* transcript might have weakened the mechanisms against chilling injury in some vegetative tissues.

Compared to Sh-13, line Sl-2 exhibited higher ectopic *CBF1* expression and PCI incidence in fruit, along with severe dwarfism and reduced photosynthetic performance in seedlings. Elevated *CBF1* expression can be beneficial in vegetative tissues, but in excess, it may suppress the plant’s capacity to cope with cold stress. Further, CBF role in the cold response might be tissue-specific and controlled by different mechanisms. While it minimizes photoinhibition and oxidative damage in vegetative tissues, it enhances fruit senescence to facilitate seed dispersal.

## 5 Conclusions

Postharvest chilling injury (PCI) is a complex physiological disorder that leads to quantitative and qualitative losses. Knowledge of the CBF1 pathway in the cold-sensitive tomato and molecular mechanisms involved in the development of cold tolerance is needed to develop effective solutions; however, it is still limited compared with that in the cold-tolerant Arabidopsis.

In the current study, transgenic tomato lines overexpressing the *CBF1* gene from *Solanum habrochaites* (*ShCBF1*) and cultivated tomato (*SlCBF1*) driven by the *RD29A* promoter, were generated. Ectopic *CBF1* expression data by RT-qPCR confirmed its induction in fruit during cold storage and upregulation for up to one week. Transgenic fruit showed aggravation of PCI symptoms, failure to ripen normally, reduced soluble solids content, abnormal volatile profile and accelerated decay when compared with wild-type fruit, and this correlated with transgenic *CBF1* expression. Transcriptomic data suggested that *CBF1* overexpression in the transgenic lines led to alterations in the ripening process and biotic and abiotic stress responses compared to WT cold stored fruit. Wild-type seedlings originating from chilled fruit displayed signs of acclimation relative to non-chilled samples. Transgenic seedlings photosynthetic performance differed between genotypes, and additional studies are needed to determine their response to cold stress in relation to wild-type seedlings.

## Data availability statement

The data presented in the study are deposited in the NCBI BioProject database, accession number PRJNA1088148.

## Author contributions

KA: Writing – original draft, Visualization, Validation, Methodology, Investigation, Formal analysis, Writing – review & editing, Resources, Funding acquisition, Conceptualization. JZ: Writing – review & editing, Methodology, Investigation, Formal analysis. FZ: Methodology, Investigation, Formal analysis, Writing – review & editing. JG: Funding acquisition, Methodology, Investigation, Formal analysis, Writing – review & editing. MW: Writing – review & editing, Methodology, Investigation, Formal analysis. DB: Writing – original draft, Supervision, Resources, Project administration, Funding acquisition, Formal analysis, Conceptualization, Writing – review & editing.
