# Supplementary material for: Ectopic overexpression of ShCBF1 and SlCBF1 in tomato suggests an alternative view of fruit responses to chilling stress postharvest
Source: Front Plant Sci. 2024 Aug 5;15:1429321. doi: 10.3389/fpls.2024.1429321 (PMC11331401; doi:10.3389/fpls.2024.1429321)
Supplement: Supplementary file 1 [file DataSheet_1.pdf]

Supplementary Material

1 Supplementary Data

Supplementary Table S1. List of primer used in this study

Supplementary Table S2. Fruit shape index (FSI) of tomato fruit

Supplementary Table S3. Photosynthetic parameters measured in wild-type seedlings stored at 2.5°C

Supplementary Table S4. Photosynthetic parameters measured in wild-type seedlings stored at 0°C

Supplementary Table S5. Photosynthetic parameters measured in transgenic seedlings stored at 0°C

Supplementary Table S6. List of VOCs identified by SPME/GC-MS

Supplementary Table S7. List of the highest differentially expressed genes (DEGs) identified by RNASeq

Supplementary Figure S1. Relative *CBF1* expression in transgenic and wild-type cold-stored seedlings.

Supplementary Figure S2. Relative *CBF1* expression of transgenic or wild-type fruit

Supplementary Figure S3. PCI symptomatology in transgenic tomato fruit after rewarming

Supplementary Figure S4. Appearance of wild-type and transgenic lines at reproductive stage

Supplementary Figure S5. Appearance of transgenic tomato plants after cold storage

Supplementary Figure S6. Amino acid sequence alignment and phylogenetic analysis of CBF proteins

Supplementary Figure S7. Relative abundance of volatile compounds without statistical significance across genotypes.

Supplementary Figure S8. Venn diagram of up- and downregulated genes in Sh-13 and Sl-2 fruit

2 Supplementary Figures and Tables

2.1 Supplementary Tables

Supplementary Table S1. List of primer used in this study.

GenBank Accession no.	Forward primer sequence (5'-3')	Reverse primer sequence (5'-3')	Fragment length (bp) (no restriction sites)	PCR product
**CLONING**				
CS191722.1	ATATTT<u>CCTGCAGG</u>GCTTGGTTGCTATGGTAGGGACT (*Sbf*I site underlined)	<u>TAGGTTTCGAAGATATTCAT</u>TTTCCAATAGAAGTAATCAAACCCTTT (*ShCBF1* fragment underlined)	1,627	*RD29A* promoter region with 20 bp overlapping *ShCBF1*
KX890304.1	GCTTGGTTGCTATGGTAGGGACT	CCC<u>AAGCTT</u>GGGTTAGATAGAATAATTCCATAAAG (*Hin*dIII site underlined)	668	*ShCBF1* coding region, longest open reading frame
-	ATATTT<u>CCTGCAGG</u>GCTTGGTTGCTATGGTAGGGACT (*Sbf*I site underlined)	CCC<u>AAGCTT</u>GGGTTAGATAGAATAATTCCATAAAG (*Hin*dIII site underlined)	2,276	Assembly between *RD29A* and *ShCBF1*
CS191722.1	ATATTT<u>CCTGCAGG</u>GCTTGGTTGCTATGGTAGGGACT (*Sbf*I site underlined)	<u>TAGGTTTCAAAGATATTCAT</u>TTTCCAATAGAAGTAATCAAACCCTTT (*SlCBF1* fragment underlined)	1,627	*RD29A* promoter region with 20 bp overlapping *SlCBF1*
NM_001247194.2	ATGAATATCTTTGAAACCTATTATTC	CCC<u>AAGCTT</u>GGGTTAGATAGAATAATTCCATAAA (*Hin*dIII site underlined)	633	*SlCBF1* coding region, longest open reading frame
-	ATATTT<u>CCTGCAGG</u>GCTTGGTTGCTATGGTAGGGACT (*Sbf*I site underlined)	CCC<u>AAGCTT</u>GGGTTAGATAGAATAATTCCATAAA (*Hin*dIII site underlined)	2,240	Assembly between *RD29A* and *SlCBF1*
**RT-qPCR**				
NM_001308447.1	GCTATCCAGGCTGTGCTTTC	CAGTAAGGTCACGACCAGCA	157	*ACT7* transcript
NM_001247194.2	AGGGGAATCAGGAAGAGGAA	ACAAGCAGAACGGCCTCTTA	156	*SlCBF1* transcript
-	GAAGACAAGGATTTGGCTTGGT	CAAACAAGCAGAACGGCCTC	100	Conserved region between *SlCBF1* and *ShCBF1* transcripts

Supplementary Table S2. Fruit shape index (FSP) of *SlCBF1*- and *ShCBF1*-overexpression lines, and wild-type ('WT') tomato fruit. Values (mean ± SE) obtained from 100 fruit per genotype. Statistical significance determined by unpaired *t*-test.

Genotype	FSP Mean ± SE	Shape	Significance when compared to WT
WT	1.42 ± 0.03	Moderately elongated	
**Sh-13**	**1.40 ± 0.03**	**Moderately elongated**	
Sh-36	1.55 ± 0.05	Moderately elongated	*
**Sl-2**	**1.41 ± 0.03**	**Moderately elongated**	
Sl-12	1.10 ± 0.03	Moderately elongated	***

Supplementary Table S3. Photosynthetic parameters measured in wild-type seedlings stored at 2.5°C for up to 72 hours. Seeds were extracted from 'chilled' and 'non-chilled fruit'. Values (mean ± SE) obtained from 16 seedlings.

**Parameter**	**Treatment**	**24h**	**48h**	**72h**
PAR (photons * m^{-2} * s^{-1})	Non-chilled	406.8 ± 24.7	438.5 ± 14.0	504.8 ± 16.2
	**Chilled**	**376 ± 21.1**	**453.7 ± 19.5**	**535.9 ± 11.0**
LEF	Non-chilled	44.9 ± 2.2	43.5 ± 2.5	33.9 ± 2.6
	**Chilled**	**50.7 ± 2.4**	**50.3 ± 3.1**	**43.1 ± 4.8**
NPQt (unitless)	Non-chilled	2.3 ± 0.3	3.6 ± 0.4	5.9 ± 0.6
	**Chilled**	**3.4 ± 0.6**	**3.5 ± 0.6**	**6.6 ± 0.9**
Phi2 (%)	Non-chilled	24.8 ± 1.6	22.3 ± 1.4	14.8 ± 0.9
	**Chilled**	**30.8 ± 2.7**	**25.1 ± 1.7**	**18.5 ± 2.5**
PhiNO (ratio)	Non-chilled	0.27 ± 0.03	0.2 ± 0.02	0.14 ± 0.01
	**Chilled**	**0.20 ± 0.03**	**0.22 ± 0.03**	**0.13 ± 0.01**
PhiNPQ (ratio)	Non-chilled	0.51 ± 0.02	0.58 ± 0.02	0.71 ± 0.01
	**Chilled**	**0.49 ± 0.04**	**0.53 ± 0.03**	**0.69 ± 0.03**
Relative chlorophyll (unitless)	Non-chilled	44.8 ± 2.2	48.1 ± 1.5	49.6 ± 1.0
	**Chilled**	**47.9 ± 1.3**	**47.9 ± 0.9**	**47.5 ± 0.9**

Supplementary Table S4. Photosynthetic parameters measured in wild-type seedlings stored at 0°C for up to 72 hours. Seeds were extracted from ‘chilled’ fruit stored at 2.5°C for three weeks, and ‘non-chilled fruit’. Values (mean ± SE) obtained from 16 seedlings. Asterisks and red letters indicate significant difference ($p < 0.05$) compared to non-chilled sample at the same time point.

Parameter	Treatment	24h	48h	72h
PAR (photons * m^{-2} * s^{-1})	Non-chilled	449.1 ± 14.6	416.9 ± 4.1	480.8 ± 9.1
	Chilled	458.3 ± 2.4	428.6 ± 12.1	490.5 ± 4.4
LEF	Non-chilled	19.2 ± 1.2	14.8 ± 1.0	11.2 ± 0.7
	Chilled	20.4 ± 1.2	14.8 ± 1.5	7.5 ± 0.7***
NPQt (unitless)	Non-chilled	5.3 ± 0.7	8.5 ± 1.1	14.7 ± 2.2
	Chilled	7.1 ± 0.7	11.7 ± 1.5	25.7 ± 2.7**
Phi2 (%)	Non-chilled	9.6 ± 0.5	7.9 ± 0.66	5.2 ± 0.3
	Chilled	9.9 ± 0.6	7.8 ± 0.9	3.4 ± 0.3***
PhiNO (ratio)	Non-chilled	0.19 ± 0.03	0.13 ± 0.02	0.08 ± 0.01
	Chilled	0.12 ± 0.01*	0.11 ± 0.03	0.05 ± 0.01*
PhiNPQ (ratio)	Non-chilled	0.71 ± 0.03	0.79 ± 0.02	0.87 ± 0.01
	Chilled	0.78 ± 0.02	0.82 ± 0.03	0.92 ± 0.01**
Relative chlorophyll (unitless)	Non-chilled	52.7 ± 0.7	51.9 ± 0.7	54.2 ± 1.5
	Chilled	47.5 ± 0.8***	47.2 ± 0.7***	49.5 ± 1.4*

Supplementary Table S5. Photosynthetic parameters measured in transgenic Sh-13 and Sl-2 seedlings stored at 0°C for up to 72 hours. PAR: Photosynthetically active radiation; LEF: Linear electron flow; NPQt: Estimate of non-photochemical quenching; Phi2: Efficiency of photosystem II; PhiNO: Ratio of incoming light that is lost via non-regulated processes; PhiNPQ: Ratio of incoming light that is directed toward non-photochemical quenching. Values (mean ± SE) obtained from 16 seedlings. Asterisks and red letters indicate significant differences ($p < 0.05$) between Sl-2 and Sh-13 at the same time point.

Parameter	Genotype	24h	48h	72h
PAR (photons * m^{-2} * s^{-1})	Sh-13	428.9 ± 5.9	426.6 ± 9.4	425.3 ± 7.3
	Sl-2	435.5 ± 8.1	424.6 ± 3.6	451.0 ± 16.6
LEF (photons * m^{-2} * s^{-1})	Sh-13	29.9 ± 2.5	18.3 ± 1.8	11.2 ± 1.6
	Sl-2	30.5 ± 2.0	18.9 ± 1.7	16.6 ± 1.2*
NPQt (unitless)	Sh-13	4.7 ± 0.7	11.3 ± 1.6	24.3 ± 3.4
	Sl-2	4.2 ± 0.6	13.3 ± 2.5	18.5 ± 2.0
Phi2 (%)	Sh-13	15.5 ± 1.3	9.7 ± 1.0	6.0 ± 0.9
	Sl-2	15.6 ± 1.0	10.0 ± 0.9	7.9 ± 0.7
PhiNO (ratio)	Sh-13	0.17 ± 0.01	0.09 ± 0.01	0.05 ± 0.01
	Sl-2	0.19 ± 0.02	0.08 ± 0.01	0.06 ± 0.01
PhiNPQ (ratio)	Sh-13	0.68 ± 0.02	0.81 ± 0.02	0.89 ± 0.02
	Sl-2	0.65 ± 0.03	0.82 ± 0.02	0.86 ± 0.02
Relative chlorophyll (unitless)	Sh-13	48.5 ± 1.9	47.2 ± 3.3	48.8 ± 1.8
	Sl-2	55.8 ± 2.2*	48.0 ± 4.5	53.9 ± 1.6

Supplementary Table S6. List of VOCs identified by SPME/GC-MS. Fruit were stored at 2.5°C for 14 days, and then rewarmed for three days.

VOC	Retention time (min)	Specific ion (m/z)
**methanol**	2.091	31
**2-methylbutanal**	2.241	41
**3-methylbutanal**	2.28	44
**1-chloro-pentane**	2.507	55
**2-ethyl-furan**	2.633	81
**pentanal**	2.996	44
**1-penten-3-one**	3.699	55
**2-methyl-3-pentanone**	4.402	43
**dimethyldisulfide**	4.783	94
**hexanal**	5.236	44
**3-methyl-butanenitrile**	6.531	43
**(E)-2-pentenal**	6.718	55
**3-hexanol**	9.155	59
**3-methylbutanol**	9.406	55
**(E)-2-hexenal**	9.556	41
**2-hexanol**	9.933	45
**octanal**	11.834	41
**1-octen-3-one**	12.193	55
**(Z)-2-heptenal**	12.802	41
**6-methyl-5-hepten-2-one**	13.233	43
**2-isobutylthiazole**	14.741	99
**(E)-2-octenal**	15.347	41
**trans linalool oxide**	15.594	59
**acetic acid**	15.758	43
**benzaldehyde**	16.897	106
**3,7-dimethyl-1,6-octadien-3-ol**	17.406	71
**1-p-metnthen-9-al**	18.287	94
**3-methylbutanoic acid**	18.998	60
**α-terpineol**	19.398	59
**β-damascenone**	20.852	69
**hexanoic acid**	21.069	60
**heptanoic acid**	22.222	60
**octanoic acid**	23.3	60
**nonanoic acid**	24.328	60

Supplementary Table S7. List of the highest differentially expressed genes (DEGs) identified by RNASeq. Data were pooled from Sh-13 and Sl-2 and compared to WT fruit. The raw RNASeq datasets are available in the National Center for Biotechnology Information (NCBI) BioProject database under accession number PRJNA1088148.

Time at 2.5°C	Gene ID	Description	log2 Fold Change against WT fruit	Mean read number
6 h	ARG2	ARGINASE 2	5.342	156.63
	LOC101264605	Aquaporin PIP1-7	3.221	285.08
	LOC101260464	Uncharacterized protein coding	3.048	6,962.30
	LOC101247747	Aquaporin PIP2-1	2.691	453.06
	LOC101267530	Protein LURP-one-related 14-like	2.143	710.40
	LOC101252483	AT-hook motif nuclear-localized protein 17	-4.823	45.95
	MADS-RIN-MADS-MC	RIN, RIPENING INHIBITOR	-3.780	118.68
	DDTFR18	Ripening regulated protein DDTFR18	-3.209	214.25
	LOC101263110	Acyl-CoA-binding domain-containing protein 3	-3.017	325.45
	LOC101246770	Vestitone reductase-like	-2.202	304.15
1 w	LOC112941094	Uncharacterized non-coding RNA	5.664	54.43
	LOC101248076	Uncharacterized non-coding RNA	1.872	539.74
	TLOG1	Cytokinin riboside 5'-monophosphate phosphoribohydrolase LOG1	1.593	425.66
	LOC101247353	Expansin-like B1	-8.195	93.40
	LOC112940478	Uncharacterized non-coding RNA	-7.003	83.45
	LOC101252483	AT-hook motif nuclear-localized protein 17	-4.557	246.99
	LOC104646357	Uncharacterized non-coding RNA	-2.176	216.90
	LOC101249071	Protein CutA, chloroplastic	-1.862	117.44

2.2 Supplementary Figures

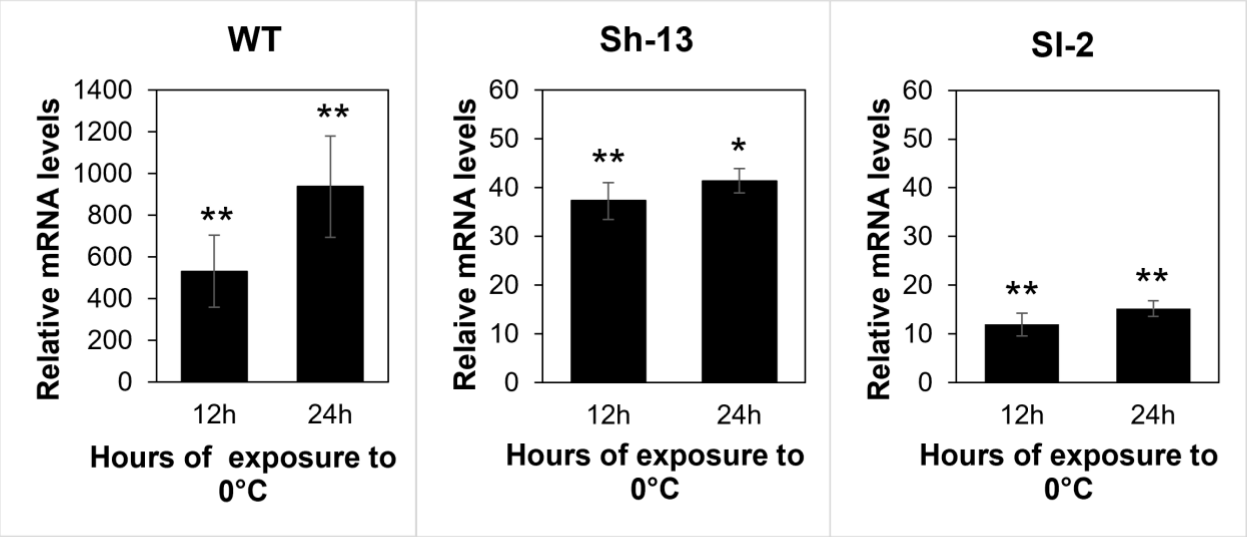

Supplementary Figure S1. Relative *CBF1* expression in transgenic and wild-type cold-stored seedlings. Seedlings were stored at 0°C for up to 24 h. *CBF1* expression was measured by quantifying both native and transgenic expression of this gene on leaf tissue by RT-qPCR. Seedlings of each genotype at room temperature were used as the calibrator for normalization. Columns with asterisks are significantly different ($p < 0.05$) to the calibrator point by unpaired *t*-test.

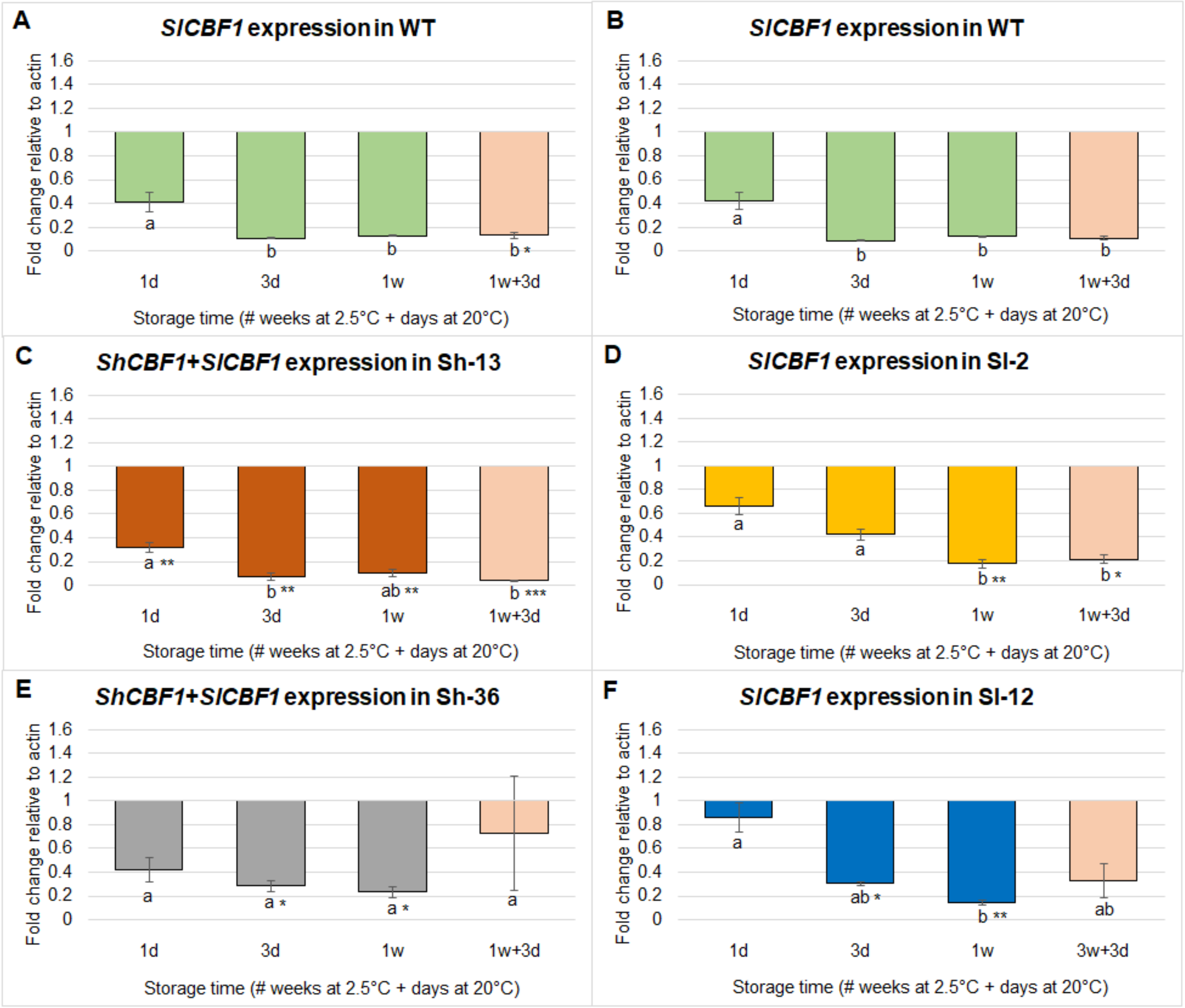

Supplementary Figure S2. Relative *CBF1* expression in transgenic and wild-type fruit stored under PCI-inducing conditions. (A, C, E) Relative expression of *SlCBF1* and *ShCBF1* in *ShCBF1*-OE and wild-type fruit. (B, D, F) Relative expression of *SlCBF1* expression in *SlCBF1*-OE and wild-type fruit. Fruit were stored at 2.5°C for up to 1 or 3 weeks, or followed by transfer to 20°C for 3 days ('1w+3d' and '3w+3d', respectively). Note: Sl-12 was measured at 3w+3d instead of at 1w+3d. Values are the mean ± SE of 18 fruit. Freshly-harvested fruit of each genotype were used as the calibrator for normalization. Different letters indicate significance among time points ($p < 0.05$) by Tukey's test. Columns with asterisks are significantly different ($p < 0.05$) to the calibrator point by unpaired *t*-test.

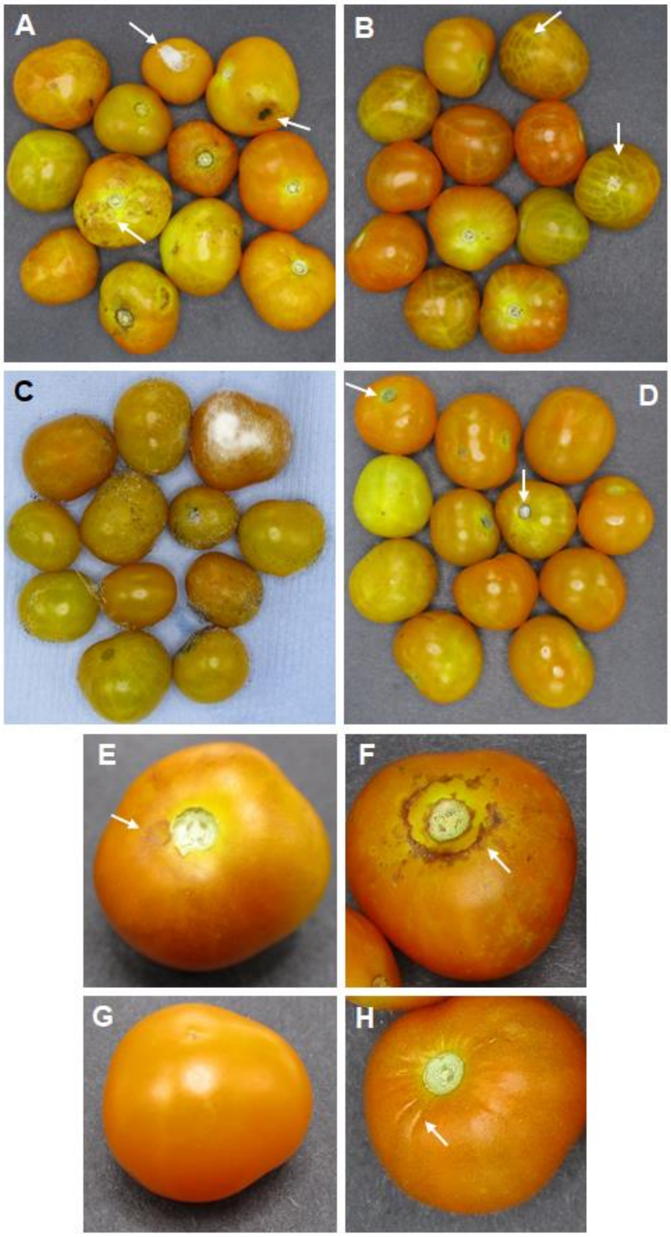

Supplementary Figure S3. PCI symptomatology in transgenic tomato fruit after 2 or 3 weeks at 2.5°C followed by RW. (A) Sl-2 fruit. White arrows indicate (i) fungal mycelia due to leaking of contents from the stem scar, (ii) decay lesion; and (iii) severe discoloration with surface pitting around stem scar. (B) Sl-12. White arrows indicate severe surface translucency with visible vascular tissue. (C) Sh-13 fruit. Fungal infestation with presence of mycelia and nesting. (D) Sl-12 fruit. Fungal growth (gray and white mold) due to leaking of contents from stem scar. (E) Sl-12 fruit. White arrow indicates mild discoloration around stem scar. (F) Sl-2 fruit. White arrow indicates severe discoloration with pitting around stem scar. (G) Sh-13 fruit with smooth, bright surface and 'swollen' appearance, highly soft to the touch, bursting easily. (H) Sh-36 fruit. White arrow indicates 'wrinkles' around the stem scar, a sign of water loss.

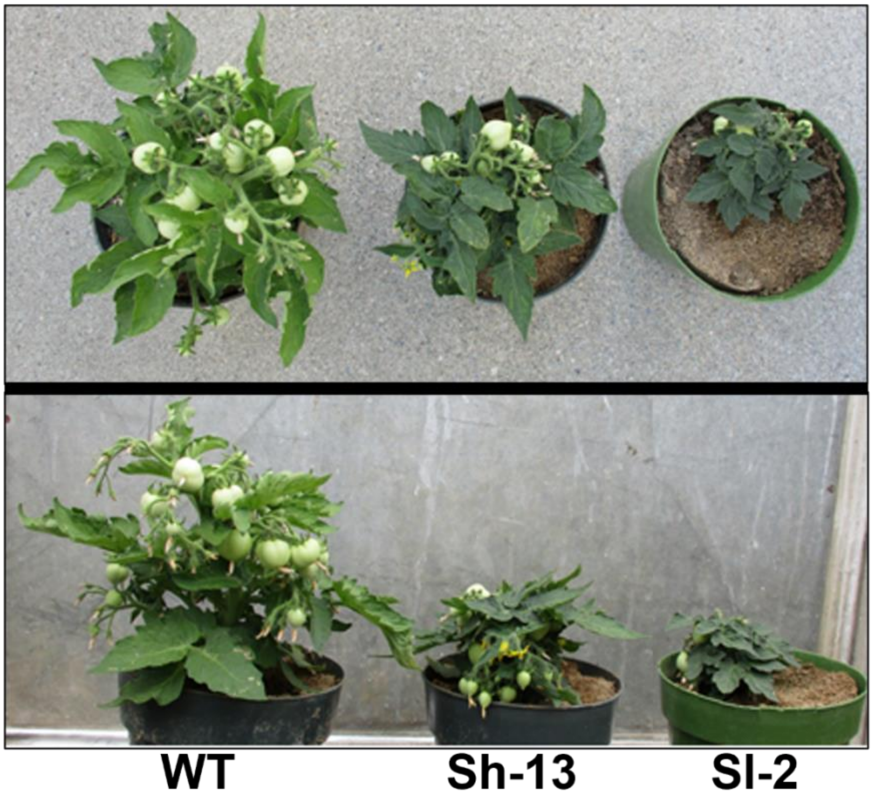

Supplementary Figure S4. Appearance of wild-type, and transgenic lines Sh-13 and Sl-2 at the reproductive stage.

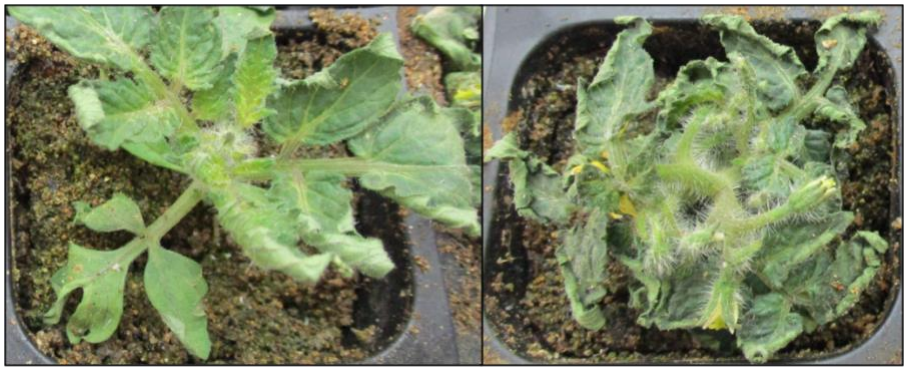

Supplementary Figure S5. Appearance of transgenic tomato plants after cold storage. Plants from lines Sh-13 and Sl-2 were stored at 0°C for 72 hours.

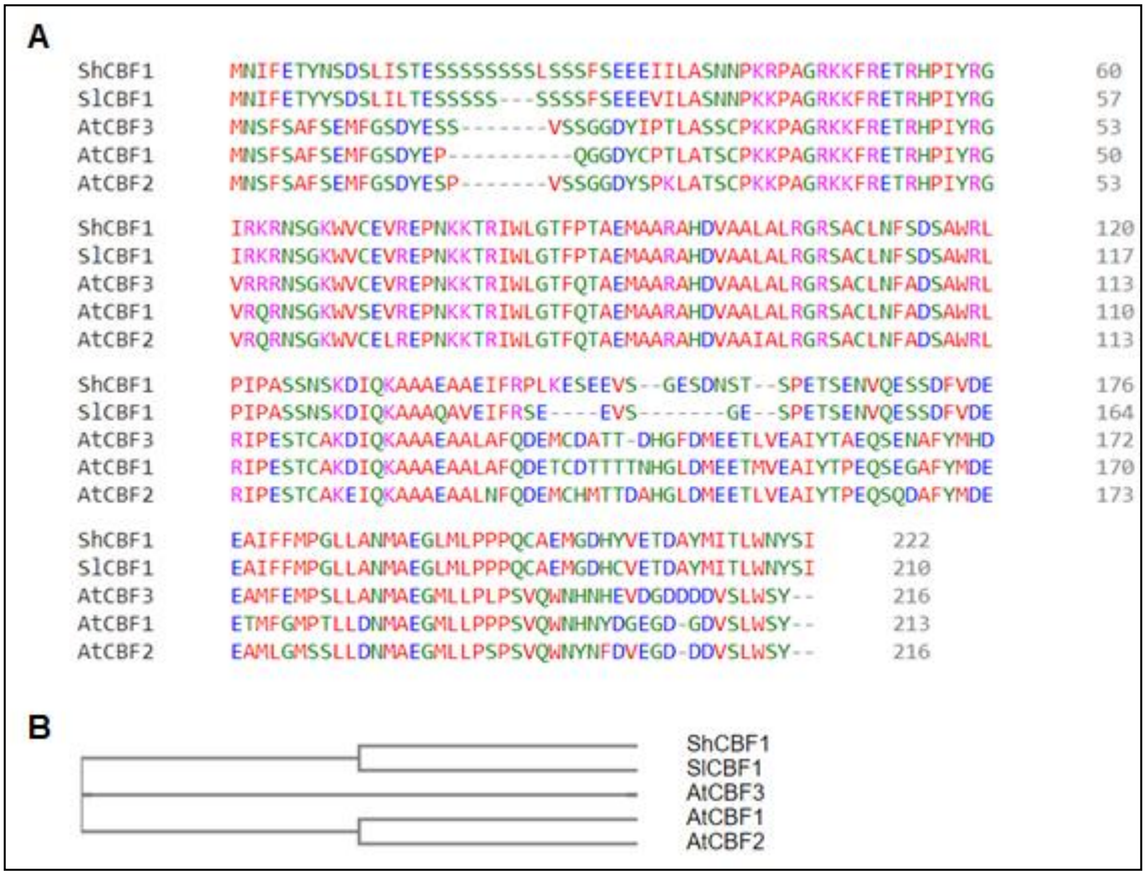

Supplementary Figure S6. Amino acid sequence alignment and phylogenetic analysis of CBF proteins. Sequences correspond to *S. habrochaites* (*ShCBF1*), *S. lycopersicum* (*SlCBF1*), and *A. thaliana* (*AtCBF1-3*). Accession numbers from GenBank are NP_567721.1 (*AtCBF1*), NP_567719.1 (*AtCBF2*), ABV27152.1 (*AtCBF3*), ARU07204.1 (*ShCBF1*) and NP_001234123.1 (*SlCBF1*). (A) Amino acid sequence alignment. Letters with the same color in a cluster indicate that the amino acid residue is conserved across sequence(s). Dashed areas in one sequence indicate absence of amino acid (s) relative to the other sequence (B) Neighbor-joining tree without distance corrections of CBF proteins obtained through the Clustal Omega tool (EMBL-EBI).

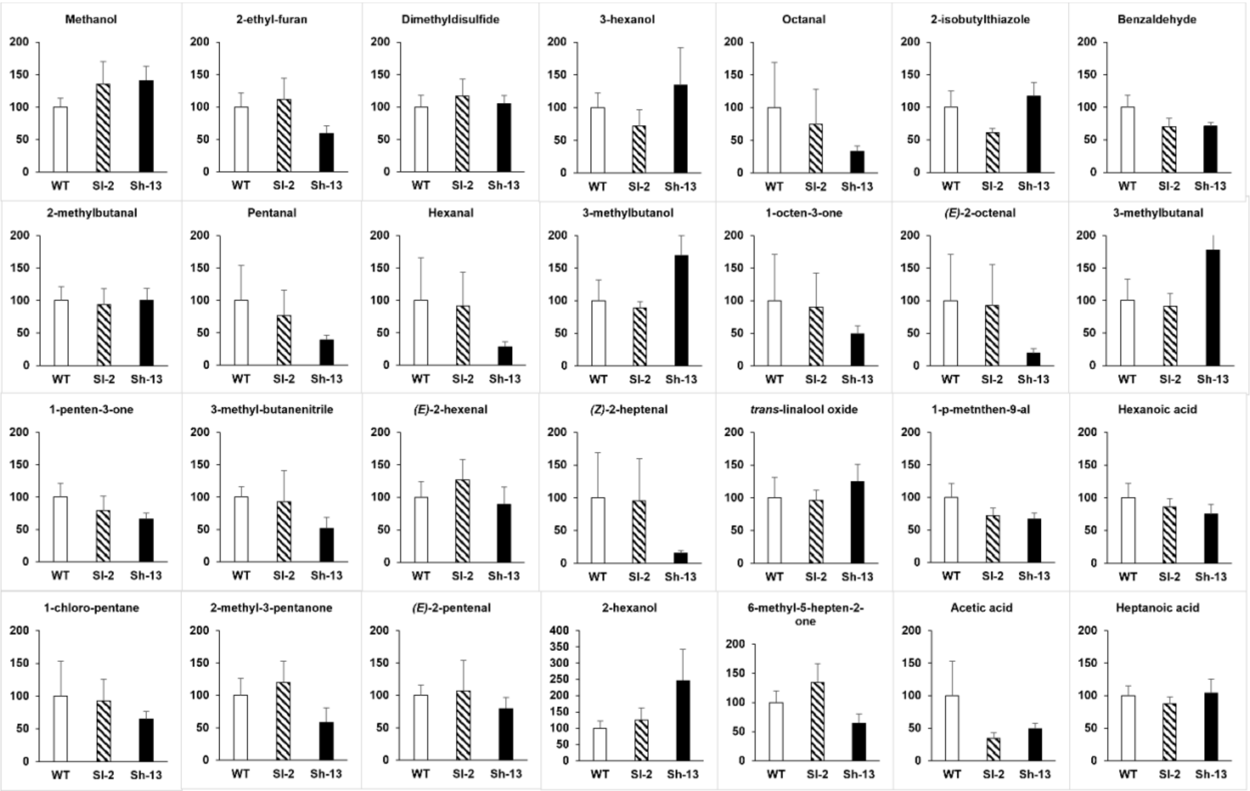

Supplementary Figure S7. Relative abundance of volatile compounds without statistical significance across genotypes. For each compound, average levels of WT were arbitrarily set to 100 for comparison. Bars are the mean ± SE of fruit.

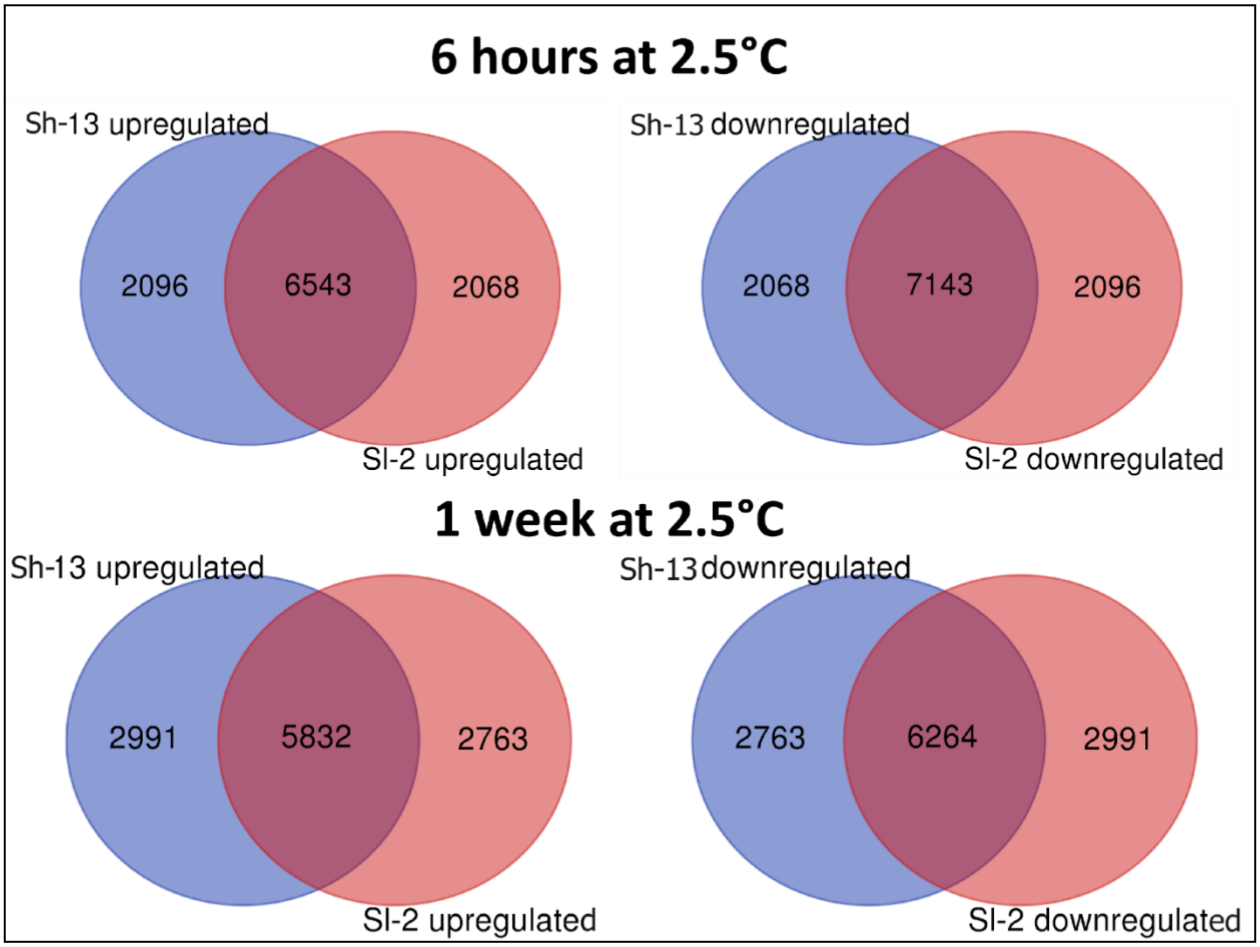

Supplementary Figure S8. Venn diagram of up- and downregulated genes in Sh-13 and Sl-2 fruit. Fruit were stored at 2.5°C for 6 hours or 1 week. Data were obtained by RNASeq analysis.
